# Centiloid cut-off values for optimal agreement between PET and CSF core AD biomarkers

**DOI:** 10.1186/s13195-019-0478-z

**Published:** 2019-03-21

**Authors:** Gemma Salvadó, José Luis Molinuevo, Anna Brugulat-Serrat, Carles Falcon, Oriol Grau-Rivera, Marc Suárez-Calvet, Javier Pavia, Aida Niñerola-Baizán, Andrés Perissinotti, Francisco Lomeña, Carolina Minguillon, Karine Fauria, Henrik Zetterberg, Kaj Blennow, Juan Domingo Gispert, for the Alzheimer’s Disease Neuroimaging Initiative, for the ALFA Study, Jordi Camí, Raffaele Cacciaglia, Marta Crous-Bou, Carme Deulofeu, Ruth Dominguez, Xavi Gotsens, Nina Gramunt, Laura Hernandez, Gema Huesa, Jordi Huguet, María León, Paula Marne, Tania Menchón, Marta Milà, Grégory Operto, Maria Pascual, Albina Polo, Sandra Pradas, Aleix Sala-Vila, Gonzalo Sánchez-Benavides, Sabrina Segundo, Anna Soteras, Laia Tenas, Marc Vilanova, Natalia Vilor-Tejedor

**Affiliations:** 1 Barcelonaβeta Brain Research Center (BBRC), Pasqual Maragall Foundation, Wellington 30, 08005 Barcelona, Spain; 2 CIBER Fragilidad y Envejecimiento Saludable (CIBERFES), Madrid, Spain; 3 Universitat Pompeu Fabra, Barcelona, Spain; 4 CIBER de Bioengeniería, Biomateriales y Nanomedicina, Madrid, Spain; 5 Nuclear Medicine Department, Hospital Clínic, Barcelona, Spain; 6 Instititut d’Investigacions Biomèdiques August Pi i Sunyer, Barcelona, Spain; 7 Clinical Neurochemistry Laboratory, Sahlgrenska University Hospital, Mölndal, Sweden; 8 Department of Psychiatry and Neurochemistry, Institute of Neuroscience and Physiology, Sahlgrenska Academy at University of Gothenburg, Sahlgrenska University Hospital, Mölndal, Sweden; 9 Department of Neurodegenerative Disease, UCL Institute of Neurology, Queen Square, London, UK; 10 UK Dementia Research Institute at UCL, London, UK

**Keywords:** AD pathophysiology, Biomarker concordance, Threshold, Positivity, Preclinical, Early detection, Positron emission tomography, Phosphorylated tau, Biomarker categorization

## Abstract

**Background:**

The Centiloid scale has been developed to standardize measurements of amyloid PET imaging. Reference cut-off values of this continuous measurement enable the consistent operationalization of decision-making for multicentre research studies and clinical trials. In this study, we aimed at deriving reference Centiloid thresholds that maximize the agreement against core Alzheimer’s disease (AD) cerebrospinal fluid (CSF) biomarkers in two large independent cohorts.

**Methods:**

A total of 516 participants of the ALFA+ Study (*N* = 205) and ADNI (*N* = 311) underwent amyloid PET imaging ([^18^F]flutemetamol and [^18^F]florbetapir, respectively) and core AD CSF biomarker determination using Elecsys® tests. Tracer uptake was quantified in Centiloid units (CL). Optimal Centiloid cut-offs were sought that maximize the agreement between PET and dichotomous determinations based on CSF levels of Aβ_42_, tTau, pTau, and their ratios, using pre-established reference cut-off values. To this end, a receiver operating characteristic analysis (ROC) was conducted, and Centiloid cut-offs were calculated as those that maximized the Youden’s J Index or the overall percentage agreement recorded.

**Results:**

All Centiloid cut-offs fell within the range of 25–35, except for CSF Aβ_42_ that rendered an optimal cut-off value of 12 CL. As expected, the agreement of tau/Aβ_42_ ratios was higher than that of CSF Aβ_42_. Centiloid cut-off robustness was confirmed even when established in an independent cohort and against variations of CSF cut-offs.

**Conclusions:**

A cut-off of 12 CL matches previously reported values derived against postmortem measures of AD neuropathology. Together with these previous findings, our results flag two relevant inflection points that would serve as boundary of different stages of amyloid pathology: one around 12 CL that marks the transition from the absence of pathology to subtle pathology and another one around 30 CL indicating the presence of established pathology. The derivation of robust and generalizable cut-offs for core AD biomarkers requires cohorts with adequate representation of intermediate levels.

**Trial registration:**

ALFA+ Study, NCT02485730

ALFA PET Sub-study, NCT02685969

**Electronic supplementary material:**

The online version of this article (10.1186/s13195-019-0478-z) contains supplementary material, which is available to authorized users.

## Introduction

Aggregation of β-amyloid (Aβ) is a neuropathological hallmark of Alzheimer disease (AD) and occurs decades before the onset of clinical symptoms occur [1, 2]. Both amyloid positron emission tomography (PET) and cerebrospinal fluid (CSF) Aβ_42_ measurement are established biomarkers of Aβ deposition that highly correlate with post-mortem [3, 4] and brain biopsy findings [5] and serving as in vivo proxies of AD pathological findings that can be assessed in vivo. They are included as part of the biological definition of AD in the recent NIA-AA 2018 research framework [6] for the definition of preclinical stages of AD [7] and as well as inclusion criteria in clinical trials [8]. CSF Aβ_42_ and amyloid PET show a high degree of agreement [9–19], even though they probably measure two different pools of amyloid. While the signal detected by amyloid PET may reflect fibrillary amyloid [20], the decrease of CSF Aβ_42_ levels more likely reflects both fibrillar and non-fibrillar Aβ deposits. Another difference is that CSF Aβ_42_ may become abnormal before amyloid PET [21, 22], while amyloid PET has been suggested to be superior for grading early symptomatic AD stages [19].

For diagnostic purposes, three ^18^F-labelled PET radiotracers have been granted marketing authorizations and are being used: [^18^F]flutemetamol (Vizamyl; GE Healthcare), [^18^F]florbetaben (Neuraceq; Life Molecular Imaging), and [^18^F]florbetapir (Amyvid, Eli Lilly). In the clinical setting, PET scans are visually read by trained specialists and are categorized as either positive or negative [23]. For quantitative purposes, the three different tracers show considerable variability when measured using the typical standardized update value ratios (SUVRs). To improve the comparability of the retention measurements across tracers, the Centiloid method has been proposed [24]. This method linearly scales the measurement of a particular tracer from 0 to 100 scale, where ‘0’ represents the average uptake in young controls and ‘100’ corresponds to the average uptake in typical AD patients at the dementia stage. To apply the Centiloid conversion, reference datasets, quantification pipelines, and regions of interest and reference are available publically from http://www.gaain.org/centiloid-project. When expressed in Centiloids (CL), optimal thresholds for positivity against visual reads typically fall within the range between 25 and 35 CL [25–27].

The applicability of CSF Aβ_42_ determinations with enzyme-linked immunosorbent assays (ELISAs) has been limited by several preanalytical and analytical factors, resulting in lot-to-lot and between-laboratory variability. These issues are expected to be improved by the availability of certified reference materials [28], and the problem with analytical variation is expected to be overcome with fully automated systems, such as the novel Elecsys® CSF immunoassay [29]. Using this system, core AD biomarkers in CSF have been compared to Aβ PET [22, 30, 31] and a CSF cut-off against PET visual read has been established by receiver operating characteristic (ROC) analysis and validated against an independent sample. The resulting areas under the curve (AUC) of CSF Aβ_42_ against Aβ PET visual read ranged from 0.85 to 0.92. Interestingly, in these studies, tau/Aβ_42_ ratios showed a higher agreement against PET visual reading (AUCs 0.94–0.96) than CSF Aβ_42_ alone.

During the last years, investigators have started interventions before the onset of clinical symptoms, when Aβ_42_ changes are detectable using CSF and amyloid PET biomarkers [32–34]. Amyloid positivity is often recognized as the earliest detectable pathophysiological abnormality in AD. Typically, positivity has been operationalized as a positive visual read in an amyloid PET scan. Accordingly, quantitative cut-offs for PET imaging, but also for CSF biomarkers, have been derived against visual reads. However, the question remains of whether lower quantitative cut-offs can be used to detect more subtle amyloid alterations with higher sensitivity, but which still provide good specificity. Such cut-offs are critical for the operationalization of preventive interventions like recruiting cognitively unimpaired individuals into prevention clinical trials. Therefore, there is a need to establish sensitive, reliable and generalizable cut-off values for amyloid PET to detect early amyloid deposition and operationalize decision-making in preventive intervention. In addition, visual inspection of PET scans can render both positive and negative reads throughout a wide range of CL values.

Initial studies to find optimal thresholds have been performed in populations recruited from clinical populations. This translates into samples with extreme values of both CSF Aβ_42_ and amyloid PET, which is AD patients with high amyloid load vs normal cognitive with low amyloid load. This approximation results in defining an optimal cut-off based on a population with low number of individuals with intermediate values around putative threshold values, which may hamper rendering. A critical consideration when deriving such cut-offs is to appropriately populate amyloid values around the cut-offs to derive optimal and robust values. On the other hand, as CSF Aβ_42_ levels start changing earlier than the PET signal, derivation of CL cut-off values against CSF in populations with initial amyloid abnormalities brings the opportunity to derive more sensitive, yet robust and generalizable, CL values associated to early amyloid accumulation.

In this study, we aimed at deriving optimal Centiloid threshold values in amyloid PET that maximize the agreement against established thresholds of CSF core AD biomarkers. To this end, we capitalized on the ALFA+ cohort of cognitively unimpaired individuals enriched for risk factors for AD [35], and in order to improve the generalizability of our results, analogous data from the Alzheimer’s Disease Neuroimaging Initiative (ADNI; http://adni.loni.usc.edu/) was pooled with that originated in the ALFA+ cohort.

## Methods

### Participants

ALFA+ is a nested longitudinal long-term study of the ALFA (for ALzheimer’s and FAmilies) cohort [35]. In brief, the ALFA cohort was established as a research platform to characterize preclinical AD in 2743 cognitively preserved individuals, aged between 45 and 75 years old with increased risk for AD. In the nested ALFA+ study, participants undergo advanced protocols of magnetic resonance imaging (MRI), amyloid PET imaging with [^18^F]flutemetamol and CSF core AD biomarkers [35]. The first consecutive 205 participants of the ALFA+ study were included in this work.

In order to have generalizable results reflecting the whole AD continuum, 311 participants from ADNI were also included in this study selected according to the following inclusion criteria: (1) AD CSF core biomarkers analysed with the Elecsys® tests available, (2) amyloid PET scan acquired in less than a year from CSF collection available, and (3) MRI acquired with a difference from the time of the PET acquisition of less than a year. All ADNI PET images included were acquired with [^18^F]florbetapir.

### CSF preanalytics of ALFA+ participants

Fresh CSF samples were collected in 15-mL polypropylene tubes (Sarstedt catalog #62.554), the supernatant aliquoted into 0.5-mL polypropylene tubes (Sarstedt catalog #72.730.005), and frozen within 2 h after lumbar puncture. Aliquots were placed into long-term storage boxes and stored at − 80 °C until shipment on dry ice for analysis.

### CSF analyses on ALFA+ and ADNI

CSF samples were measured using the Elecsys® β-amyloid(1–42) [29], and the Elecsys® phosphotau (181P) and Elecsys® total-tau immunoassays for CSF on a cobas e 601 analyzer (software version 05.02) at the Clinical Neurochemistry Laboratory, University of Gothenburg, Sweden (ALFA+) or at the Biomarker Research Laboratory, University of Pennsylvania, USA (ADNI), according to the kit manufacturer’s instructions and as described in previous studies [29].

### Predefined CSF cut-offs against PET visual read

The BioFINDER and ADNI CSF AD core biomarker cut-offs were previously determined against amyloid PET visual read classification [22, 30]. ADNI participants were categorized using ADNI-specific CSF thresholds (please see Additional file 1: Table S1). Given that the ALFA+ study shares the same preanalytical and analytical conditions as BioFINDER, we used the same thresholds previously published for BioFINDER to categorize ALFA+ participants (please, see Additional file 1: Table S1).

On the other hand, we used the previously described conversion factor from BioFINDER to ADNI values [30] in order to pool herein the ALFA+ values with those of ADNI (only for figures, not used for CL cut-offs derivation).

### Neuroimage acquisition procedures

Each cohort had its own acquisition protocol. For ALFA+, a T1-weighted MRI and an [^18^F]flutemetamol PET scan was acquired in all participants (mean time difference 97.1 days; range [14–343]). The T1-weighted 3D-TFE sequence was acquired in a Philips 3 T Ingenia CX scanner with a voxel size of 0.75 × 0.75 × 0.75 mm^3^, FOV 240 × 240 × 180 mm^3^, sagittal acquisition, flip angle 8°, TR = 9 .9ms, TE = 4 .6ms, TI = 900 ms. PET imaging was conducted in a Siemens Biograph mCT, following a cranial CT scan for attenuation correction. Participants were injected with 185 MBq (range 166.5–203.5 Mbq) of [^18^F]flutemetamol, and 4 frames of 5 min each were acquired 90 min post-injection. Images were reconstructed with an OSEM3D algorithm using 8 iterations and 21 subsets and with point spread function (PSF) and time of flight (TOF) corrections into a matrix size of 1.02 × 1.02 × 2.03 mm.

The methods for ADNI PET and MRI acquisition methods are described in more detail elsewhere (http://adni.loni.usc.edu/methods/documents/). In brief, all PET images were acquired with [^18^F]florbetapir, which consisted of 4 frames of 5 min each, acquired at 50 to 70 min post injection. Most of the T1 sequences used for normalization were magnetization-prepared rapid acquisition gradient echo (MPRAGE), acquired with 1 .5T or 3 T scanners. All images, ALFA+ and ADNI, were visually inspected for quality control.

### Image processing

All PET images were preprocessed following the Centiloid [24] pipeline using SPM12 (https://www.fil.ion.ucl.ac.uk/spm/software/spm12/). In brief, PET frames were coregistered. Averaged images were then coregistered to corresponding MRI scans. MRIs were then segmented and normalized to the MNI space together with PET images. We calculated the SUVr in MNI space using the target region provided in the GAAIN website (www.gaain.org) and the whole cerebellum as reference region. SUVr values were then transformed to the Centiloid scale as explained in Additional file 1: Supplementary methods.

### Statistical analysis

Demographic characteristics of both cohorts were first compared. *T* test for independent samples was used with continuous variables and *χ*^2^ with dichotomous variables. To be able to directly compare Aβ_42_ measures from both cohorts, we had to transform ADNI data to account for pre-analytical conditions [30]. This transformation was only used to perform scatter plots but not to perform any other analysis, as each dataset had their own cut-offs for the CSF biomarkers (Additional file 1: Table S1).

Optimal Centiloid cut-offs were calculated as those that maximized the Youden’s J Index (YI) or the overall percentage agreement (OPA; “accuracy”). YI statistic consists of the summation of the sensitivity and specificity [36], and the OPA reflects the percentage of cases with concordant binary classification with CSF and PET. Both were calculated on the pooled ALFA+ and ADNI data as a function of Centiloid values for Aβ_42_, phosphorylated tau (pTau), total tau (tTau), and pTau/Aβ_42_ and tTau/Aβ_42_ ratios. For each CSF biomarker as binary outcome, optimal cut-offs were selected as those showing the maximum value of one of the statistics after minimally smoothing true-positive, true-negative, false-positive and false-negative curves using the ‘smooth’ function in Matlab (v2018b) with the ‘lowess’ method and a span value of 0.1.

On top of YI and OPA, we also calculated the positive percentage agreement (PPA, “sensitivity”) and negative percentage agreement (NPA, “specificity”) and the area under the curve (AUC) of the receiver operating characteristic (ROC) analysis. All 95% confidence intervals (95% CI) for all statistics were derived using bootstrapping methods (*n* = 5000).

In order to explore the generalizability of the calculated thresholds, we also derived them in each cohort individually. Robustness of the Centiloid cut-offs were assessed by deriving them against more liberal CSF thresholds (higher for Aβ_42_ and lower for tau, Additional file 1: Table S1). With this new CSF categorization, we wanted to include those participants that are close to the threshold but still classified as negative (“grey zone”, [GZ]). To calculate these new CSF thresholds, we add (or subtract) the 10% of the original threshold, as this slight variation in the CSF thresholds showed helping to reduce false negatives and to be strongly associated with future Aβ_42_ positivity [37, 38].

## Results

### Demographic characteristics and CSF and amyloid biomarkers

Table 1 shows demographics and characteristics of CSF and amyloid PET biomarkers in the two cohorts included, i.e. ALFA+ and ADNI, which have some differences. The ALFA+ cohort has younger participants, more women and significantly less proportion of positive participants on all AD CSF core biomarkers, as expected as it includes only cognitively preserved participants. By contrast, ADNI participants are in more advanced stages of the disease, with higher number of *APOE-ε4* carriers and higher frequency of positive core AD CSF biomarkers (Table 1). This also translates into a significant difference in both the mean and average of amyloid PET CL values between both cohorts. As shown in Figs. 2a, 3a and 4a, the ALFA+ cohort covers intermediate CSF and CL values, whereas ADNI participants’ CSF and CL measures show a more bimodal pattern. Mean and SD values for CSF biomarkers and Centiloid measures can be found in Additional file 1: Table S2. Scatterplots for pTau and tTau can be found in Additional file 1: Figure S1.

**Table 1:** Demographics and characteristics of CSF biomarkers and PET quantification measures, overall and by cohort. All the characteristics shown in this table were statistically different (*p* < 0.001) between cohorts

	ALL (*n* = 516)	ALFA+ (*n* = 205)	ADNI (*n* = 311)
Age, mean (SD) [range]	69.13 (9.10) [50–92]	61.01 (4.85) [50–74]	74.48 (7.07) [56–92]
Women, *n* (%)	286 (55.4)	134 (65.4)	152 (48.9)
Education, years mean (SD)	14.98 (3.37)	13.49 (3.58)	15.96 (2.82)
*APOE-ε4* carriers, *n* (%)	260 (50.4)	81 (39.5)	179 (57.6)
Positive Aβ_42_, *n* (%)	273 (52.9)	60 (29.3)	213 (68.5)
Positive pTau, *n* (%)	323 (62.6)	56 (27.3)	267 (85.9)
Positive tTau, *n* (%)	294 (57.0)	50 (24.4)	244 (78.5)
Positive pTau/Aβ_42_, *n* (%)	258 (50.0)	24 (11.7)	234 (75.2)
Positive tTau/Aβ_42_, *n* (%)	246 (47.7)	21 (10.2)	225 (72.3)
Diagnostic, *n* (%) CN/MCI/AD	256 / 237 / 23 (49.6)/(45.9)/(4.5)	205 / 0 / 0 (100)/(0)/(0)	36 / 237 / 23 (11.6)/(76.2)/(7.4)
Time difference CSF-PET, days mean (SD) [range]	45.2 (60.3) [0–343]	97.1 (65.1) [14–343]	11.0 (17.2) [0–126]

*Aβ β*-amyloid, *AD* Alzheimer’s disease, *ADNI* Alzheimer’s Disease Neuroimaging Initiative, *APOE* Apoliprotein E, *CN* cognitively normal, *MCI* mild cognitive impaired participants, *pTau* phosphorylated tau, *SD* standard deviation, *tTau* total tau

### Optimal CL cut-offs

We computed the optimal CL cut-off values to differentiate individuals within the Alzheimer’s *continuum* and controls using the AD CSF core biomarkers as a reference. We performed the analysis with CSF Aβ_42_ alone, pTau/Aβ_42_ and tTau/Aβ_42_ ratios and also pTau and tTau alone. We studied the pooled data from ALFA+ and ADNI cohorts, using cohort-specific CSF thresholds.

**Fig. 1:**
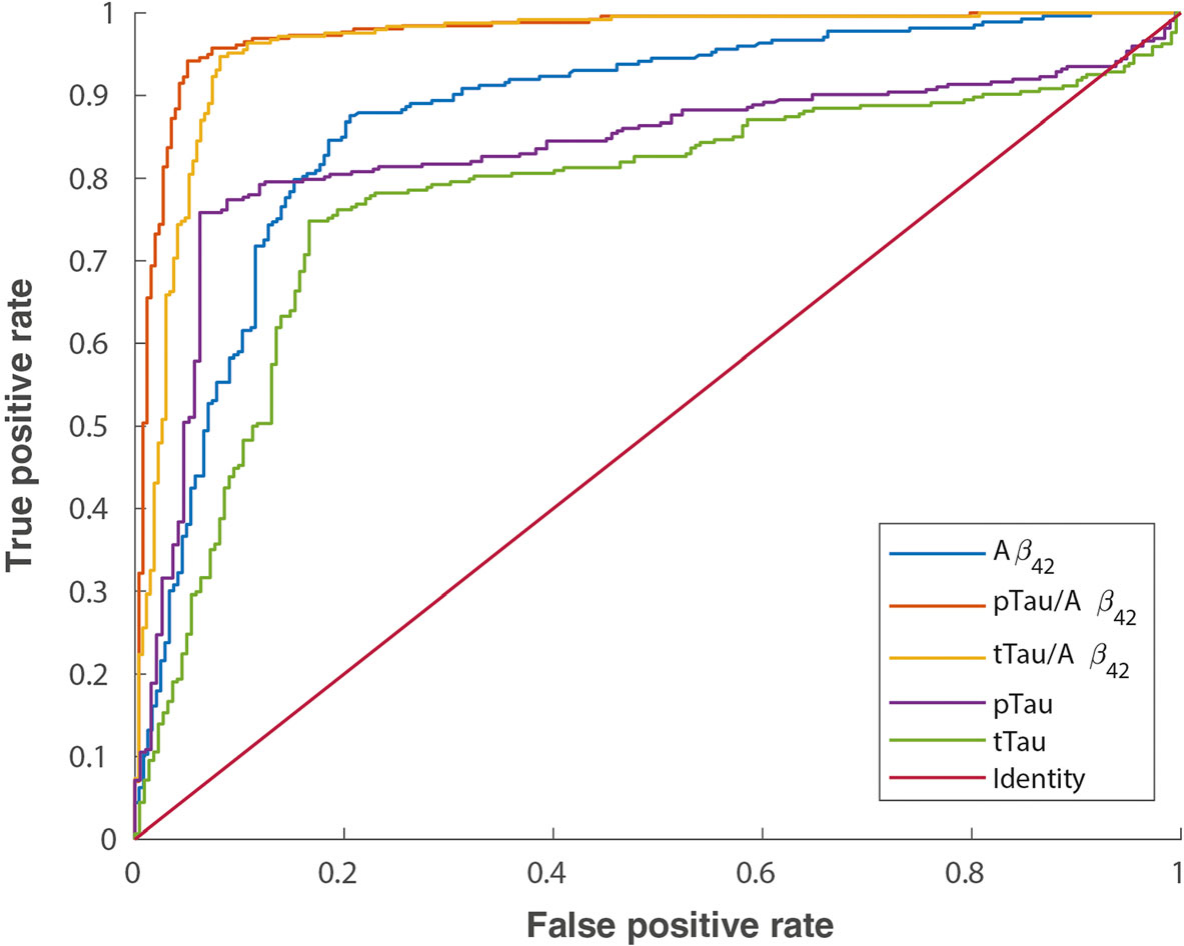
Summary of ROC curves for Centiloid cut-off derivation against CSF biomarkers. pTau, phosphorylated tau; tTau, total tau; CSF, cerebrospinal fluid; ROC, receiver operating characteristic

Table 2 summarizes the optimal CL values, using the pooled data from ALFA+ and ADNI cohorts, and associated statistical performance against CSF Aβ_42_, pTau/Aβ_42_, tTau/Aβ_42_, pTau and tTau. Figure 1 shows the associated ROC curves.

**Table 2:** Centiloid cut-off against CSF biomarkers. Derivation was done by maximization of YI or OPA. Other statistics for this cut-off have been also derived: PPA, NPA and AUC. 95% CI are shown between brackets. All participants’ information was used to derive these cut-offs

Biomarker		YI’s derived cut-offs					OPA’s derived cut-offs				
	AUC	CL cut-off	YI^a^	OPA	PPA	NPA	CL cut-off	YI	OPA^a^	PPA	NPA
Aβ_42_	0.874 [0.840–0.903]	12.1	0.659 [0.594–0.721]	0.831 [0.798–0.861]	0.852 [0.809–0.892]	0.806 [0.753–0.851]	11.6	0.659 [0.593–0.720]	0.831 [0.798–0.861]	0.855 [0.812–0.895]	0.804 [0.750–0.849]
pTau/Aβ_42_	0.974 [0.956–0.9985]	28.8	0.886 [0.842–0.922]	0.943 [0.921–0.961]	0.941 [0.904–0.964]	0.945 [0.915–0.970]	28.8	0.886 [0.842–0.922]	0.943 [0.921–0.961]	0.941 [0.904–0.964]	0.945 [0.915–0.970]
tTau/Aβ_42_	0.961 [0.940–0.975]	29.7	0.863 [0.818–0.905]	0.931 [0.908–0.952]	0.948 [0.912–0.970]	0.915 [0.882–0.946]	30.1	0.863 [0.818–0.905]	0.931 [0.908–0.952]	0.948 [0.911–0.970]	0.915 [0.882–0.947]
pTau	0.833 [0.793–0.869]	29.3	0.689 [0.629–0.745]	0.822 [0.787–0.853]	0.755 [0.704–0.798]	0.934 [0.898–0.966]	18.7	0.680 [0.610–0.732]	0.823 [0.787–0.852]	0.773 [0.726–0.816]	0.907 [0.856–0.939]
tTau	0.774 [0.727–0.814]	28.6	0.573 [0.505–0.640]	0.781 [0.747–0.815]	0.745 [0.693–0.792]	0.828 [0.779–0.875]	26.7	0.573 [0.502–0.639]	0.781 [0.745–0.815]	0.748 [0.697–0.795]	0.825 [0.773–0.870]

^a^Shows the statistic was used to derive each cut-off

*pTau* phosphorylated tau, *tTau* total tau, *CL* Centiloids, *OPA* overall percentage agreement (“accuracy”), *YI* Youden’s J Index, *PPA* positive percentage agreement (“sensitivity”), *NPA* negative percentage agreement (“specificity”), *AUC* area under the curve

### CSF Aβ_42_

**Fig. 2:**
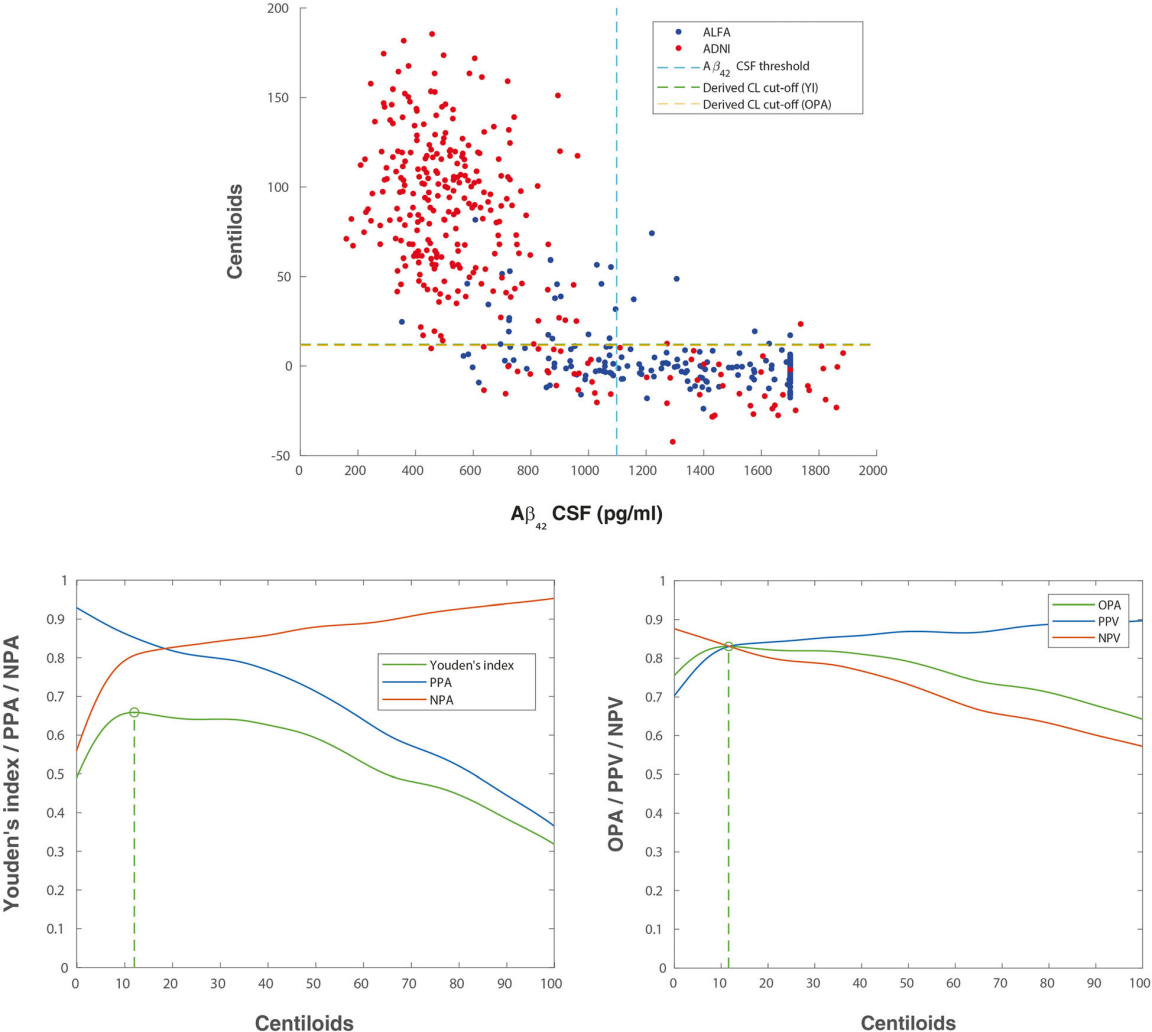
Derivation of amyloid PET Centiloid cut-offs using Aβ_42_ CSF biomarker. Upper row shows a scatterplot of PET quantitative measures in Centiloids against CSF Aβ_42_ biomarker values for both ALFA+ (blue) and ADNI (red) participants. Vertical lines depict previously published CSF threshold (Aβ_42_ 1098 pg/ml [22, 30]), and horizontal lines depict optimal cut-offs derived in this study (YI derived: green, OPA derived: yellow). Two outliers were excluded of this picture for having CL value higher than 200. Second row shows this cut-offs derivation by the maximization of YI (left) and OPA (right). The YI (green) was calculated from PPA (blue) and NPA (red) values. The OPA (green) was calculated from PPV (blue) and NPV (red) values. Derivation was done with participants of both cohorts merged. Aβ, amyloid; CSF, cerebrospinal fluid; YI, Youden’s J index; PPA, positive percentage agreement; NPA, negative percentage agreement; CL, Centiloids

Derivation of Centiloid cut-off against Aβ_42_ is shown in Fig. 2. The resulting cut-offs with this analysis were 12.1 CL with YI’s optimization and 11.6 CL OPA’s optimizations, which corresponded with maximum values YI of 0.66 (95% CI 0.59–0.72) and OPA of 0.83 (95% CI 0.81–0.86), respectively. The corresponding area under the curve for CSF Aβ_42_ was of 0.87 (95% CI 0.84–0.90). PPA and NPA values for these CL cut-offs are shown in Table 2.

The optimal cut-offs calculated against 10% variation of the CSF threshold were similar (CL = 11.1 CL with OPA, 0.85, and CL = 12.9 with YI, 0.70; Additional file 1: Table S3 and Figure S2), and the AUC was slightly improved AUC = 0.90. When CL cut-offs were derived separately in the two independent cohorts, the AUC for CSF Aβ_42_ was higher in the ADNI cohort than in ALFA+ (0.85 vs 0.76 Additional file 1: Table S4). The optimal threshold for the ALFA+ cohort were slightly lower to the one found in the pooled analysis (CL = 5.4 with YI and CL = 10.7 with OPA, Additional file 1: Table S4 and Figure S3), whereas, for ADNI, the optimal cut-off resulted was significantly higher (CL = 36.2 CL with YI and CL = 33.1 with OPA, Additional file 1: Table S4 and Figure S4).

### CSF tau/Aβ_42_ ratios and tau

**Fig. 3:**
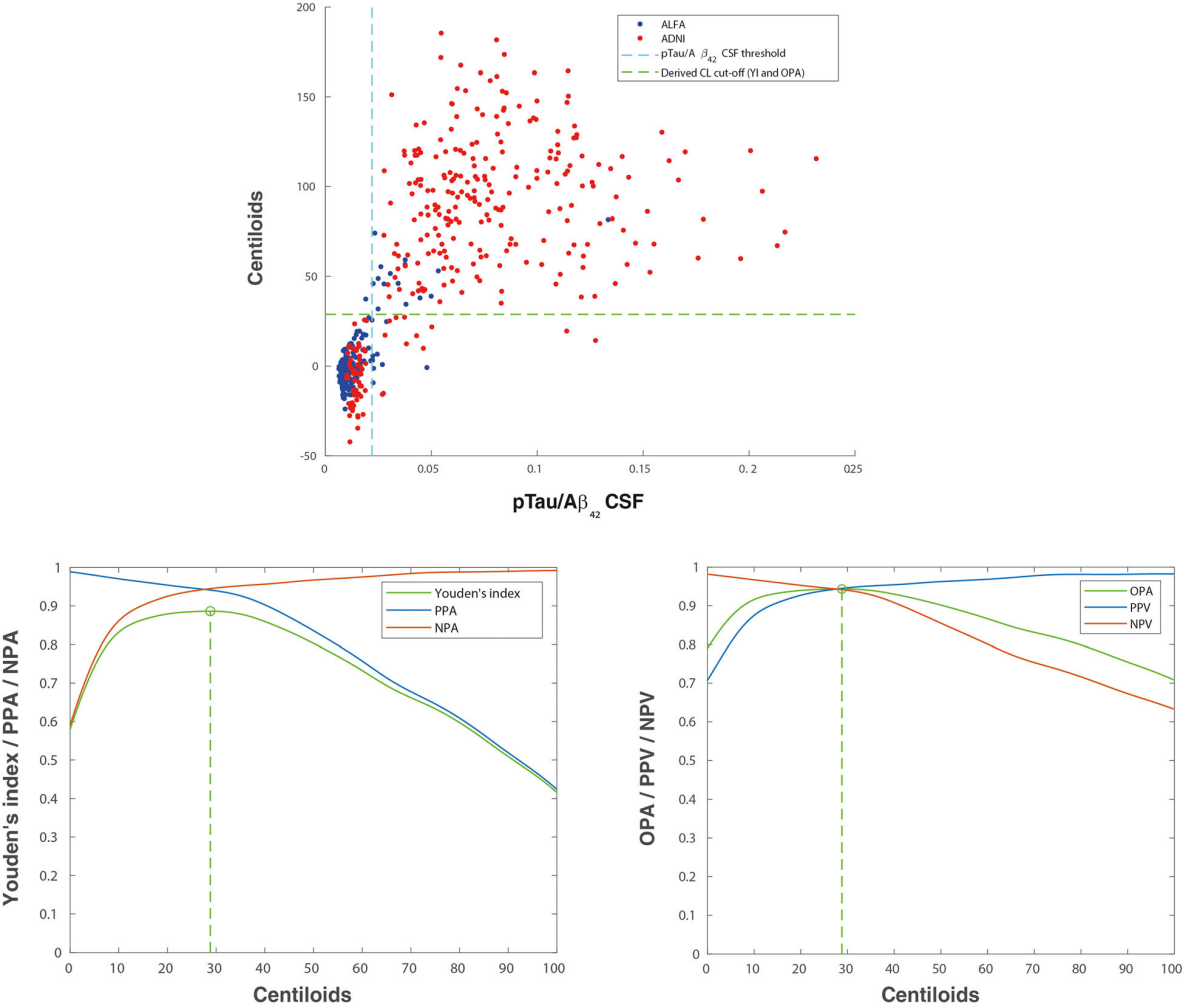
Derivation of amyloid PET Centiloid cut-offs for CSF pTau/Aβ_42_. Top row shows scatterplots of PET quantitative measures in Centiloids against CSF pTau/Aβ_42_ biomarker values for both ALFA+ (blue) and ADNI (red) participants. Vertical line depicts previously published CSF thresholds (pTau/Aβ_42_ 0.022 [22, 30]), and horizontal line depicts optimal cut-offs derived in this study (YI and OPA derived cut-offs are equal). Two outliers were excluded of this picture for having CL value higher than 200. Second row shows this cut-off derivation by maximization of YI (left) and OPA (right). The YI (green) was calculated from PPA (blue) and NPA (red) values. The OPA (green) was calculated from PPV (blue) and NPV (red) values. Derivation was done with participants of both cohorts merged. pTau, phosphorylated tau; CSF, cerebrospinal fluid; YI, Youden’s J index; PPA, positive percentage agreement; NPA, negative percentage agreement; CL, Centiloids

**Fig. 4:**
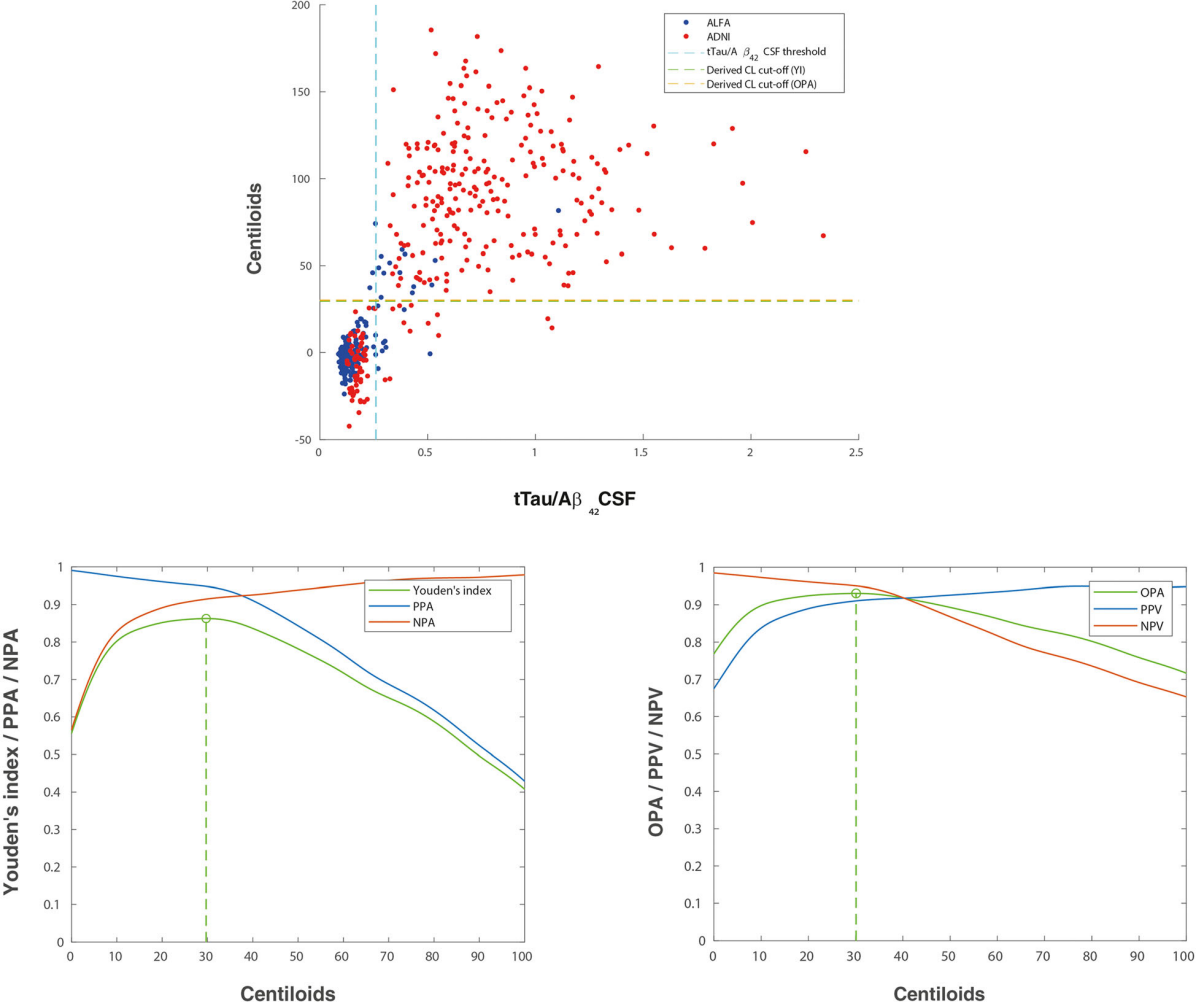
Derivation of amyloid PET Centiloid cut-offs for CSF tTau/Aβ_42_. Top row shows scatterplots of PET quantitative measures in Centiloids against CSF tTau/Aβ_42_ biomarker values for both ALFA+ (blue) and ADNI (red) participants. Vertical line depicts previously published CSF thresholds (tTau/Aβ_42_ 0.26 [22, 30]), and horizontal lines depict optimal cut-offs derived in this study (YI derived: green, OPA derived: yellow). Two outliers were excluded of this picture for having CL value higher than 200. Second row shows this cut-off derivation by maximization of YI (left) and OPA (right). The YI (green) was calculated from PPA (blue) and NPA (red) values. The OPA (green) was calculated from PPV (blue) and NPV (red) values. Derivation was done with participants of both cohorts merged. tTau, total tau; CSF, cerebrospinal fluid; YI, Youden’s J index; PPA, positive percentage agreement; NPA, negative percentage agreement; CL, Centiloids

Very similar results were found in both tau over Aβ_42_ ratios (Figs. 3 and 4). The optimal cut-off derived with both cohorts merged for pTau/Aβ_42_ was 28.8 CL with an AUC of 0.97 [0.96–0.99] with both YI and OPA’s maximization and for tTau/Aβ_42_ 29.7CL with YI’s maximization and 30.1 CL with OPA’s maximization with an AUC of 0.96 [0.94–0.97] (Table 2).

Unlike for CSF Aβ_42_, optimal thresholds against variations of the CSF cut-offs resulted in a reduced optimal threshold of CL = 21.4 for both tau ratios with YI’s maximization and CL = 20.6 for both tau ratios with OPA’s maximization (Additional file 1: Table S3 and Figure S2). When derived in both cohorts separately, thresholds were again slightly lower in the ALFA+ cohort (CL = 20.0 and CL = 24.8 for pTau ratio and CL = 20.1 and CL = 24.9 for tTau ratio; Additional file 1: Figure S3 and Table S4) and significantly higher for ADNI (CL = 34.4 and CL = 31.5 for pTau ratio and CL = 34.7 and CL = 32.5 for tTau ratio; Additional file 1: Figure S4 and Table S4).

Optimal cut-offs for CSF pTau and tTau were similar to those for the ratios, but with lower AUCs (Table 2). Meanwhile, tTau cut-offs remained relatively stable when using YI or OPA as criterion (CL = 28.6 and CL = 28.8, respectively); pTau cut-offs changed highly (29.3 CL with YI vs 18.7 with OPA; Additional file 1: Table S3). The analysis against 10% CSF variations resulted in similar cut-offs with similar AUCs except for tTau cut-off resulting from OPA’s maximization that lowered up to 15.9 CL (Additional file 1: Table S3 and Figure S2). Finally, the behaviour of the cut-offs in the two cohorts separately was quite different with respect to tau over Aβ_42_ ratios and resulted in lower AUCs than the optimal cut-off (Additional file 1: Figure S2, S3 and, Table S4).

## Discussion

In this paper, we sought to calculate the optimal Centiloid cut-off values from amyloid PET data to maximize the agreement against previously established thresholds for positivity on core AD CSF biomarkers. At a first glance, this might be regarded as a circular exercise, since these CSF cut-offs were originally derived to maximally concord with positive visual reading of PET scans. Under this rationale, all resulting Centiloid cut-offs would have be expected to fall in the range that optimally discriminates negative from positive visual reads, which is between 25 and 35 CL [25, 26]. On the contrary, optimal agreement for CSF Aβ_42_ was observed for a cut-off of 12 CL. This seemingly unexpected result can be explained by the clearly non-linear relationship between amyloid PET Centiloids and CSF Aβ_42_, as previously reported [39]. Almost all subjects with CSF Aβ_42_ over 1000 pg/ml showed Centiloid values below 20, and only for CSF values < 1000 pg/ml, a linear association could be intuited. This nonlinear association makes goodness criteria (both the Youden’s Index and the overall percentage agreement) to plateau between 10 and 40 CL (Fig. 2). Under these circumstances, to derive stable optimal cut-offs, it is critical to make use of a test sample comprising both sufficient concordant positive and negative cases as well as a good representation of individuals falling within intermediate amyloid ranges (10 < CL < 40 and 500 < CSF Aβ_42_ < 1000 pg/ml). In this study, this was achieved by pooling the ADNI and ALFA+ datasets.

The independent derivation of optimal CSF Aβ_42_ cut-offs in the two cohorts confirmed this rationale. In the ADNI sample, optimal values fell in the expected range of visual reads (between 33 and 36 CL) whereas the optimal threshold in the ALFA+ sample is closer to that in the pooled sample (between 5 and 11 CL). This result confirms that the derivation of optimal cut-offs is very sensitive to the recruitment strategy of the reference sample, with clinical ones rendering higher cut-offs than population based ones with a better representation around the range of values where cut-offs are expected to lay. Previous literature has suggested that CSF Aβ_42_ values become positive before amyloid PET [21, 22]. This may be related to amyloid PET visual read and SUVRs cut-offs on clinical populations. In these populations, the visual read is performed in patients with either prodromal AD or dementia due to AD; hence, the amyloid load is supposed to have reached its ceiling. By contrast, the ALFA+ population reflects a cohort of early amyloid accumulators at risk for cognitive impairment; therefore, a positive visual read may be reached when amyloid is still not at its peak. Indeed, the threshold of 12 CL is robust against variations in the cut-off for CSF positivity (Additional file 1: Table S3) as well as if only the ALFA+ dataset is used for its derivation.

Although 12 CL may initially be regarded as a low value for amyloid positivity, it matches recent reports of Centiloid cut-offs against postmortem measures of AD neuropathology showing that 12.2 CL optimally detected Consortium to Establish a Registry for Alzheimer’s Disease (CERAD) moderate-to-frequent neuritic plaques, whereas 24.4 CL identified intermediate-to-high AD neuropathologic change (ADNC) differences [40]. Another similar study showed that a threshold < 10 CL was optimal for ruling out the presence of amyloid plaques, whereas CL > 20 suggests significant amyloid pathology [26]. However, we would like to point out that we did not want to affirm that the CL cut-offs found show amyloid pathology, but only to find those CL values that maximally agree with those of the CSF core AD biomarkers. The fact that the CL cut-offs derived in this study agree with a previous one done with neuropathological data is only a marker that these values might have an actual biological meaning, more than only a practical one. But this hypothesis should be tested in another work, preferably with longitudinal data.

Unlike CSF Aβ_42_, for tau/CSF Aβ_42_ ratios, the optimal CL cut-offs fell within the expected range (28–30 CL) given the linear relationship between this biomarker and amyloid PET Centiloids. Indeed, tau/CSF Aβ_42_ ratios showed higher AUC versus amyloid PET Centiloids than CSF Aβ_42_, in agreement with the previous reports [22, 27, 30, 31]. The higher capacity of tau/CSF Aβ_42_ ratios to predict Centiloids may be accounted for by two different factors. On the one hand, CSF ratios may provide a more stable measurement than absolute values since they provide an inherent normalization against protein production and release to the CSF, between-individual variations in CSF dynamics, and pre-analytical conditions. Therefore, the lower variability in the CSF ratios may account for better AUCs. On the other hand, CSF Aβ_42_ has been proposed to become abnormal prior to amyloid PET [21, 41]. This fact might stem from the fact that both techniques probe different pools of the amyloid protein. Therefore, the combination of measurements of Aβ with those of tau, a pathological change that is expected to occur later in the AD *continuum* [41], might show better agreement with amyloid PET, which is also expected to become abnormal later than CSF Aβ_42_.

Together with previous studies, the observed thresholds might be useful to flag two different inflection points in preclinical AD stages. A cut-off below 12 CL might be optimal to rule out-amyloid pathology, whereas a cut-off over 29 CL might be denoting established pathology. These kinds of thresholds have typically been used to dichotomize continuous values into two categories for clarity and ease of use. However, alternatives have also been considered and include the score of the severity of each biomarker on a semi-continuous scale as considered, for instance, in the A/T/N scheme [42]. Therefore, an option would be to categorize the full range of variation of biomarker values in three categories: one that excludes any pathology, another intermediate category that would indicate early and developing pathology and a third one that corresponds with established pathology.

Two goodness criteria have been used here to derive optimal cut-offs: the Youden’s Index, which balances sensitivity and specificity, and the overall percentage agreement, which is sensitive to the percentages of positives versus negatives in the test sample. Both rendered very similar values and the Centiloid cut-offs proposed here are robust against variations in the threshold values for CSF positivity. Still, the Youden’s index showed a more noisy behaviour than the overall percentage agreement, particularly in the analysis of the two individual samples. In order to obtain more robust estimates of classification performance, more subjects across the full AD *continuum* would be needed. This is a relevant effect because in previous similar works, the Youden’s Index has been typically selected as the reference measurement of agreement [22, 30, 31]. Hansson et al. [30] added reliability measures to performance metrics to derive optimal cut-offs. We handled the noisy behaviour of performance metrics by deriving optimal cut-offs after some minimal smoothing of the data. This approach proved to be efficacious to derive stable cut-offs even in the analysis of the individual cohorts.

Irrespective of the approach to counter the effect of noisy agreement estimates, additional analysis with larger samples might be needed to yield more robust and generalizable cut-offs. To this end, future work will focus on pooling additional samples. In addition to a limited sample size, we rely on the comparability of PET and CSF measures across the two studied samples. While agreement on CSF data is certainly improved with the Elecsys® tests and with the use of the Centiloid method on PET scans, we cannot rule out the presence of a certain degree of sample-dependent bias in the data analysed. Still, when computing the cut-offs solely using the ALFA+ cohort results were very similar, thus suggesting that any remaining bias is small and did not have a significant impact on our results. Another limitation may stem from the somewhat limited sample analysed here may not be sufficient to derive robust generalizable cut-off values. Additional analysis with bigger sample sizes and more amyloid PET tracers that the two used here may overcome this limitation.

In summary, we have derived optimal Centiloid values to maximize the agreement against core AD CSF biomarkers. Regarding Aβ, a relatively low value of 12 CL optimally corresponded to CSF Aβ_42_ positivity, in line with Centiloid thresholds derived against post-mortem measures of AD neuropathology. On the other hand, CSF tau/Aβ_42_ ratios were better predicted by a higher Centiloid cut-off of 29 CL, which is in line with those optimally discriminating positive from negative visual reads on PET scans. In agreement with previous reports, CSF tau/Aβ_42_ ratios showed a higher capacity to predict amyloid PET Centiloids than CSF Aβ_42_. Overall, our results provide reference values in the Centiloid scale and suggest two relevant inflection points the development of early AD pathology across the full AD *continuum*.

## Additional file

Additional file 1: Supplementary data including supplementary methods and results. (DOCX 1010 kb)

## Abbreviations

AD: Alzheimer’s disease
ADNC: AD neuropathologic change
ADNI: Alzheimer’s Disease Neuroimaging Initiative
ALFA: Alzheimer and Families
AUC: Area under the curve
Aβ: β-Amyloid
CERAD: Consortium to Establish a Registry for Alzheimer’s Disease
CI: Confidence interval
CL: Centiloid
CSF: Cerebrospinal fluid
ELISA: Enzyme-linked immunosorbent assay
GZ: Grey zone
MPRAGE: Magnetization-prepared rapid acquisition gradient echo
MRI: Magnetic resonance imaging
NPA: Negative percentage agreement (“specificity”)
OPA: Overall percentage agreement (“accuracy”)
PET: Positron emission tomography
PPA: Positive percentage agreement (“sensitivity”)
PSF: Point spread function
pTau: Phosphorylated tau
ROC: Receiver operating characteristic
SUVr: Standardized uptake value ratio
TOF: Time of flight
tTau: Total tau
YI: Youden’s J index
