# Supplementary material for: Centiloid cut-off values for optimal agreement between PET and CSF core AD biomarkers
Source: Alzheimers Res Ther. 2019 Mar 21;11:27. doi: 10.1186/s13195-019-0478-z (PMC6429814; doi:10.1186/s13195-019-0478-z)
Supplement: Supplementary file 1 — Supplementary data including supplementary methods and results. (DOCX 1010 kb) [file 13195_2019_478_MOESM1_ESM.docx]

## Additional file 1: Centiloid cut-off values for optimal agreement between PET and CSF core AD biomarkers

Salvadó G, Molinuevo JL, Brugulat A, Falcon C, Grau-Rivera O, Suárez-Calvet M, Pavia J , Niñerola-Baizán A, Perissinotti A, Lomeña F, Minguillon C, Fauria K, Zetterberg H, Blennow K, and Gispert JD, for the Alzheimer’s Disease Neuroimaging Initiative (ADNI), for the ALFA Study.

### Methods

| CSF biomarker | ALFA+ cut-offs | | ADNI cut-offs | |
| --- | --- | --- | --- | --- |
|  | Positivity | Grey zone | Positivity | Grey zone |
| Aβ_42_ (pg/ml) | 1098 | 1207.8 | 880 | 968.8 |
| pTau (pg/ml) | 19.2 | 17.28 | 19.2 | 17.28 |
| tTau (pg/ml) | 242 | 217.8 | 242 | 217.8 |
| pTau/Aβ_42_ | 0.022 | 0.0198 | 0.028 | 0.0252 |
| tTau/Aβ_42_ | 0.26 | 0.234 | 0.33 | 0.297 |

**Table s1:** CSF thresholds for positivity and GZ. Thresholds for positivity were from [1,2]. Thresholds for GZ were derived by calculating the 10% of the positivity threshold and adding (or subtracting) it to the positive thresholds [3].

Abbreviations: CSF, cerebrospinal fluid; pTau, phosphorylated tau; tTau, total tau; GZ, grey zone.

To calibrate our Centiloid pipeline we followed the instructions of the reference paper [4]. In brief, we first downloaded the reference images acquired with ^11^C-PiB (34 young controls [YC] and 45 AD patients). The standard pipeline to preprocess the images as explained in [4] was followed with the only difference of using SPM12 instead of SPM8. Mean SUVr values were calculated for both groups of participants, with a result of 1.010 (0.046) as a mean (SD) value for YC and 2.076 (0.201) for AD patients. Both results fall within the 2% range accepted by the original paper (1.009 and 2.076, respectively).

We then transformed each participant SUVr to Centiloids using Eq. 2.2.1 of the original paper [4]. In our case, the final equation resulted as: CL = 100(^PiB^SUVr - 1.010) / (2.076 - 1.010). When we compared the resultant Centiloid values against the ones published, we found a high correlation (R^2^ = 0.997) with all the criteria (Slope = 0.999, Intercept = 0.048 CL) falling within the recommended range (R^2^ > 0.98, slope: 0.98 to 1.02 and intercept: -2 to 2 CL).

## As we did not use the same tracer as used in the original Centiloid paper (^11^C-PiB), we also had to calibrate the transformation of our both tracers ([^18^F]flutemetamol and [^18^F]florbetapir) to the reference (^11^C-PiB). To do so, we downloaded the two datasets aimed for this purpose on the GAAIN website (www.gaain.org/centiloid). The two groups of images were processed as previously explained and SUVr values were calculated for both ^11^C-PiB and the two new tracers. Linear regressions between [^18^F]flutemetamol vs. ^11^C-PiB, and [^18^F]florbetapir vs. ^11^C-PiB were then derived. The resulting conversion from [^18^F]flutemetamol to ^11^C-PiB was: ^PiB^SUVr = 1.289 ^FLUTE^SUVr - 0.271 (R^2^ = 0.963). And from [^18^F]florbetapir to ^11^C-PiB: ^PiB^SUVr = 1.757 ^FBP^SUVr - 0.770 (R^2^ = 0.884).

## Therefore, when we calculated SUVr values from ALFA and ADNI, we first transformed these values to ^11^C-PiB SUVr values using the linear transformations detailed above. After that, we also transform these new values to Centiloids using the aforesaid transformation (CL = 100(^PiB^SUVr - 1.010) / (2.076 - 1.010)).

### Results

|  | ALL (n=516) | ALFA+ (n=205) | ADNI (n=311) |
| --- | --- | --- | --- |
| Concentration Aβ_42_, pg/ml mean (SD) | NC | 1352.1 (358.7) | 866.2 (493.4) |
| Concentration pTau/Aβ_42_, mean (SD) | NC | 0.014 (0.011) | 0.053 (0.039) |
| Concentration tTau/Aβ_42_, mean (SD) | NC | 0.165 (0.102) | 0.528 (0.342) |
| Concentration pTau, pg/ml mean (SD) | 27.9 | 16.8 (8.3) | 35.3 (18.9) |
| Concentration tTau, pg/ml mean (SD) | 295.9 (149.8) | 205.0 (77.5) | 355.9 (155.8) |
| Centiloid, mean (SD) | 45.8 (55.6) | 2.6 (16.3) | 74.2 (54.0) |

**Table s2:** Concentration of CSF biomarkers and PET quantification measures, overall and by cohort.

Abbreviations: Aβ, amyloid; pTau, phosphorylated tau; tTau, total tau; CL, Centiloids; CSF, cerebrospinal fluid; SD, standard deviation; NC, not comparable.

|  |  | YI's derived cut-offs | | | | | OPA's derived cut-offs | | | | |
| --- | --- | --- | --- | --- | --- | --- | --- | --- | --- | --- | --- |
| Biomarker | **AUC** | **CL cut-off** | **YI*** | **OPA** | **PPA** | **NPA** | **CL**  **cut-off** | **YI** | **OPA*** | **PPA** | **NPA** |
| Aβ_42_ | 0.898  [0.866 - 0.923] | 12.90 | 0.702  [0.637 - 0.759] | 0.846  [0.779 - 0.863] | 0.823  [0.828 - 0.918] | 0.880  [0.814 - 0.875] | 11.10 | 0.701  [0.635 - 0.757] | 0.847  [0.789 - 0.871] | 0.831  [0.816 - 0.908] | 0.869  [0.815 - 0.875] |
| pTau/Aβ_42_ | 0.983  [0.966 - 0.992] | 21.40 | 0.922  [0.884 - 0.950] | 0.959  [0.905 - 0.963] | 0.936  [0.958 - 0.995] | 0.985  [0.940 - 0.974] | 20.60 | 0.922  [0.883 - 0.949] | 0.959  [0.906 - 0.964] | 0.938  [0.956 - 0.993] | 0.983  [0.939 - 0.974] |
| tTau/Aβ_42_ | 0.983  [0.966 - 0.991] | 21.40 | 0.922  [0.884 - 0.948] | 0.959  [0.905 - 0.962] | 0.936  [0.960 - 0.995] | 0.985  [0.939 - 0.973] | 20.60 | 0.922  [0.883 - 0.948] | 0.959  [0.907 - 0.963] | 0.938  [0.957 - 0.993] | 0.983  [0.939 - 0.973] |
| pTau | 0.829  [0.790 - 0.864] | 30.50 | 0.679  [0.624 - 0.735] | 0.798  [0.669 - 0.763] | 0.718  [0.925 - 0.989] | 0.961  [0.762 - 0.832] | 15.70 | 0.665  [0.603 - 0.726] | 0.803  [0.699 - 0.789] | 0.746  [0.872 - 0.956] | 0.920  [0.768 - 0.837] |
| tTau | 0.801  [0.758 - 0.837] | 29.60 | 0.625  [0.558 - 0.688] | 0.784  [0.665 - 0.763] | 0.717  [0.862 - 0.947] | 0.908  [0.748 - 0.820] | 15.90 | 0.612  [0.539 - 0.676] | 0.787  [0.695 - 0.790] | 0.743  [0.814 - 0.913] | 0.869  [0.751 - 0.822] |

**Table s3:** Centiloid cut-off against CSF biomarkers GZ thresholds. Derivation was done by maximization of YI or OPA. Other statistics for these cut-offs have been also derived: PPA, NPA and AUC. 95% CI are shown between brackets. All participants' information was used to derive these cut-offs. * Shows the statistic was used to derive each cut-off.

Abbreviations: Aβ, amyloid; pTau, phosphorylated tau; tTau, total tau; CL, Centiloids; CSF, cerebrospinal fluid; GZ, grey zone; OPA, overall percentage agreement; YI, Youden's J Index; OPA, overall percentage agreement ("accuracy"); PPA, positive percentage agreement ("sensitivity"); NPA, negative percentage agreement ("specificity"); AUC, area under the curve.

| ALFA (n=205) | | | | | | | | | | | | |
| --- | --- | --- | --- | --- | --- | --- | --- | --- | --- | --- | --- | --- |
|  |  | **YI's derived cut-offs** | | | | | | **OPA's derived cut-offs** | | | | |
| Biomarker | **AUC** | **CL**  **cut-off** | **YI*** | | **OPA** | **PPA** | **NPA** | **CL**  **cut-off** | **YI** | **OPA*** | **PPA** | **NPA** |
| Aβ_42_ | 0.760  [0.677 - 0.831] | 5.40 | 0.396  [0.275 - 0.537] | 0.770  [0.392 - 0.632] | | 0.525  [0.841 - 0.936] | 0.871  [0.725 - 0.834] | 10.70 | 0.362  [0.244 - 0.489] | 0.798  [0.290 - 0.529] | 0.400  [0.912 - 0.980] | 0.962  [0.737 - 0.844] |
| pTau/Aβ_42_ | 0.893  [0.762 - 0.958] | 20.00 | 0.681  [0.488 - 0.860] | 0.949  [0.503 - 0.874] | | 0.698  [0.955 - 0.995] | 0.983  [0.916 - 0.976] | 24.80 | 0.665  [0.461 - 0.830] | 0.954  [0.473 - 0.838] | 0.673  [0.966 - 1.001] | 0.992  [0.920 - 0.978] |
| tTau/Aβ_42_ | 0.892  [0.748 - 0.956] | 20.10 | 0.678  [0.464 - 0.862] | 0.945  [0.500 - 0.888] | | 0.706  [0.940 - 0.989] | 0.973  [0.911 - 0.972] | 24.90 | 0.659  [0.447 - 0.838] | 0.950  [0.464 - 0.856] | 0.678  [0.952 - 0.996] | 0.982  [0.915 - 0.975] |
| pTau | 0.614  [0.516 - 0.707] | 24.80 | 0.279  [0.163 - 0.404] | 0.799  [0.175 - 0.414] | | 0.289  [0.964 - 0.998] | 0.990  [0.737 - 0.847] | 29.10 | 0.275  [0.174 - 0.413] | 0.801  [0.175 - 0.415] | 0.279  [0.994 - 1.000] | 0.997  [0.745 - 0.853] |
| tTau | 0.605  [0.504 - 0.703] | 17.50 | 0.262  [0.141 - 0.406] | 0.799  [0.188 - 0.450] | | 0.303  [0.915 - 0.980] | 0.959  [0.740 - 0.847] | 28.90 | 0.25  [0.146 - 0.405] | 0.810  [0.163 - 0.422] | 0.273  [0.955 - 0.998] | 0.984  [0.756 - 0.863] |
| ADNI (n=311) | | | | | | | | | | | | |
|  |  | **YI's derived cut-offs** | | | | | | **OPA's derived cut-offs** | | | | |
| Biomarker | **AUC** | **CL**  **cut-off** | **YI*** | **OPA** | | **PPA** | **NPA** | **CL**  **cut-off** | **YI** | **OPA*** | **PPA** | **NPA** |
| Aβ_42_ | 0.850  [0.791 - 0.898] | 36.20 | 0.613  [0.509 - 0.705] | 0.861  [0.922 - 0.979] | | 0.953  [0.556 - 0.744] | 0.660  [0.818 - 0.896] | 33.10 | 0.611  [0.514 - 0.710] | 0.862  [0.929 - 0.982] | 0.960  [0.557 - 0.745] | 0.651  [0.822 - 0.900] |
| pTau/Aβ_42_ | 0.954  [0.907 - 0.978] | 34.40 | 0.813  [0.722 - 0.888] | 0.937  [0.940 - 0.986] | | 0.966  [0.753 - 0.916] | 0.847  [0.908 - 0.961] | 31.50 | 0.810  [0.726 - 0.893] | 0.938  [0.942 - 0.988] | 0.971  [0.755 - 0.918] | 0.839  [0.910 - 0.963] |
| tTau/Aβ_42_ | 0.919  [0.866 - 0.954] | 34.70 | 0.755  [0.654 - 0.837] | 0.920  [0.949 - 0.991] | | 0.973  [0.682 - 0.861] | 0.782  [0.887 - 0.948] | 32.50 | 0.753  [0.658 - 0.841] | 0.921  [0.952 - 0.992] | 0.977  [0.683 - 0.862] | 0.776  [0.888 - 0.949] |
| pTau | 0.772  [0.678 - 0.845] | 32.10 | 0.577  [0.416 - 0.704] | 0.833  [0.801 - 0.889] | | 0.851  [0.573 - 0.847] | 0.726  [0.785 - 0.872] | 4.90 | 0.454  [0.277 - 0.587] | 0.855  [0.866 - 0.936] | 0.905  [0.379 - 0.683] | 0.549  [0.810 - 0.888] |
| tTau | 0.654  [0.572 - 0.729] | 32.20 | 0.313  [0.183 - 0.443] | 0.759  [0.783 - 0.878] | | 0.837  [0.357 - 0.601] | 0.477  [0.707 - 0.802] | 5.80 | 0.226  [0.099 - 0.339] | 0.768  [0.842 - 0.921] | 0.886  [0.224 - 0.449] | 0.340  [0.716 - 0.810] |

**Table s4:** Centiloid cut-offs against main CSF biomarkers for both cohorts separately. Derivation was done by maximization of YI and OPA. Other statistics for these cut-offs have been also derived: PPA, NPA and AUC. 95% CI are shown between brackets.

Abbreviations: Aβ, amyloid; pTau, phosphorylated tau; tTau, total tau; CSF, cerebrospinal fluid; CL, Centiloids; YI, Youden's J Index; PPA, positive percentage agreement ("sensitivity"); NPA, negative percentage agreement ("specificity"); OPA, overall percentage agreement ("accuracy"); AUC, area under the curve.

**
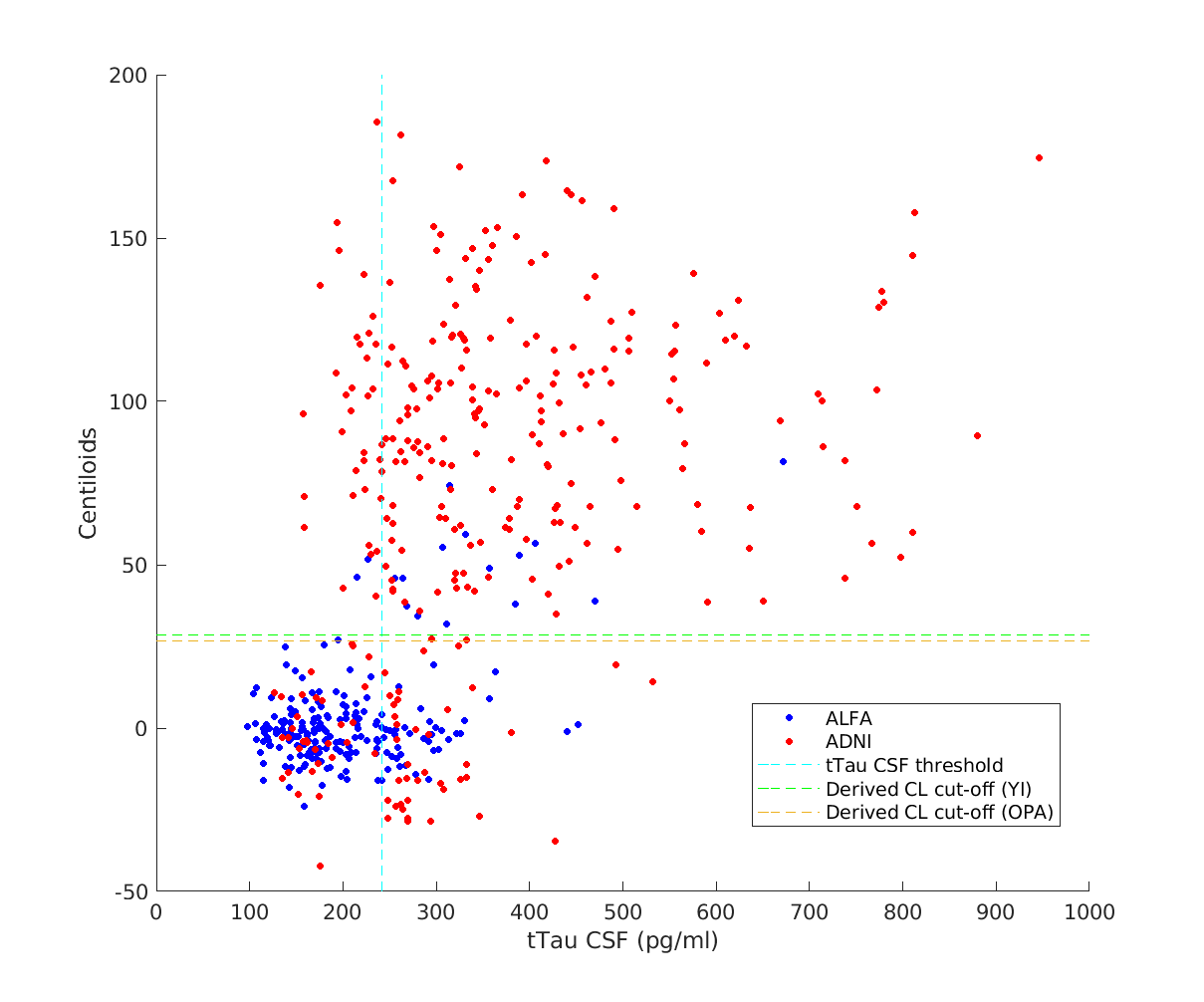

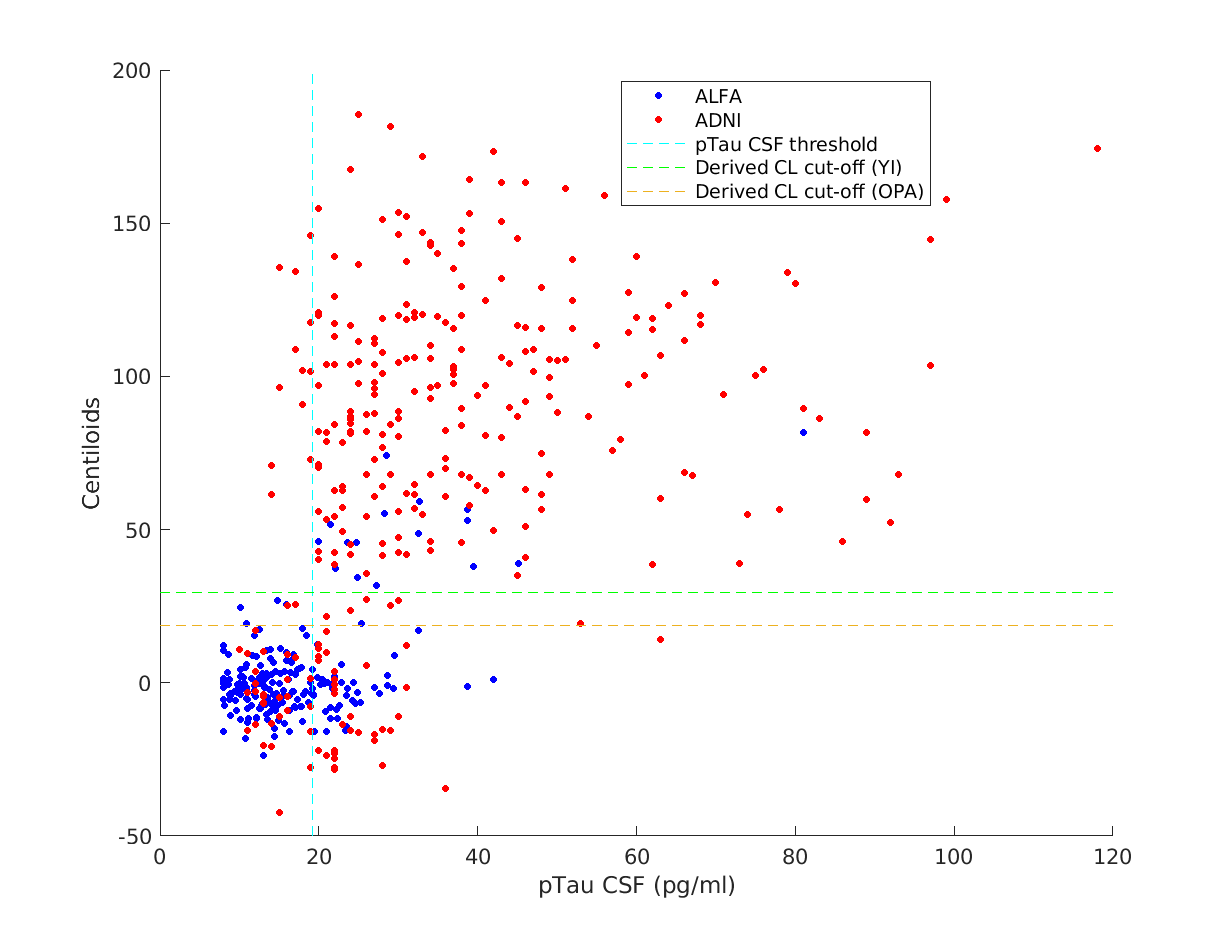
**

**Figure s1:** Scatterplots of PET quantitative measures in Centiloids against CSF biomarkers values. pTau (a) and tTau biomarkers are illustrated in the figure. Each dot represents a scan and its colour indicates the cohort (ALFA+: blue, ADNI: red). Vertical line depict previously published CSF thresholds (pTau: 19.2 pg/ml, tTau: 242 pg/ml [2]) and, horizontal lines depict optimal cut-offs derived in this work (YI derived: green, OPA derived: yellow). Two outliers were excluded of this picture for having CL value higher than 200.

Abbreviations: pTau, phosphorylated tau; tTau, total tau; CSF, cerebrospinal fluid.

|  | **Youden’s index** | **Overall percentage agreement** |
| --- | --- | --- |
| **Aβ_42_** | 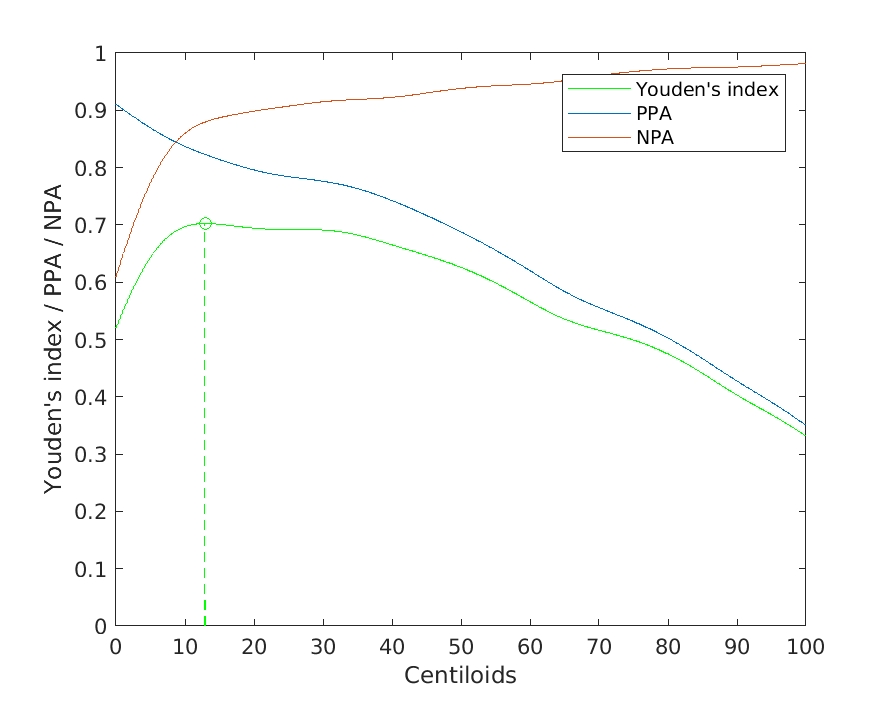 | 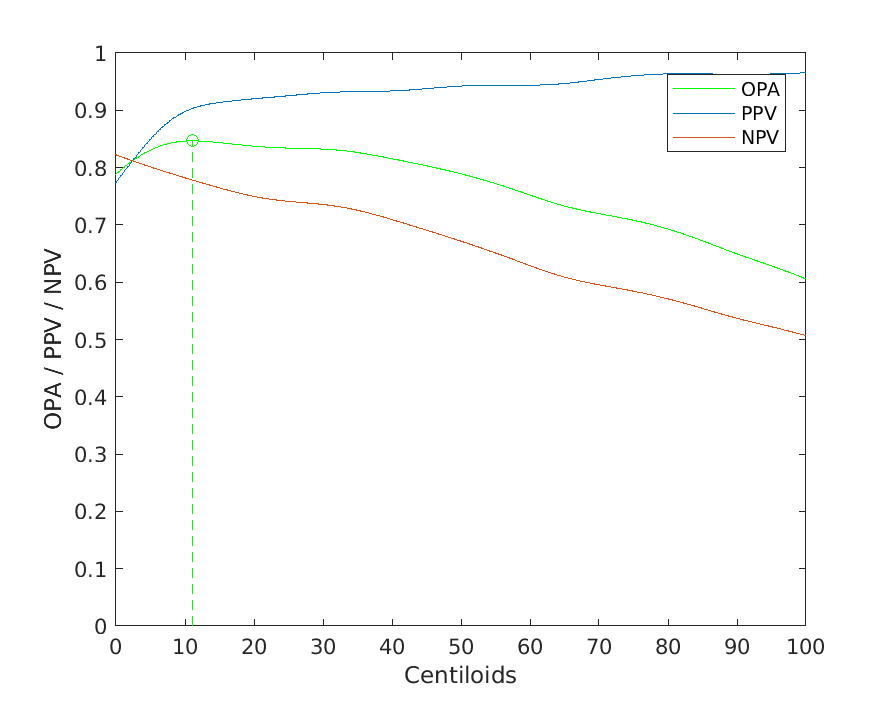 |
| **pTau/Aβ_42_** | 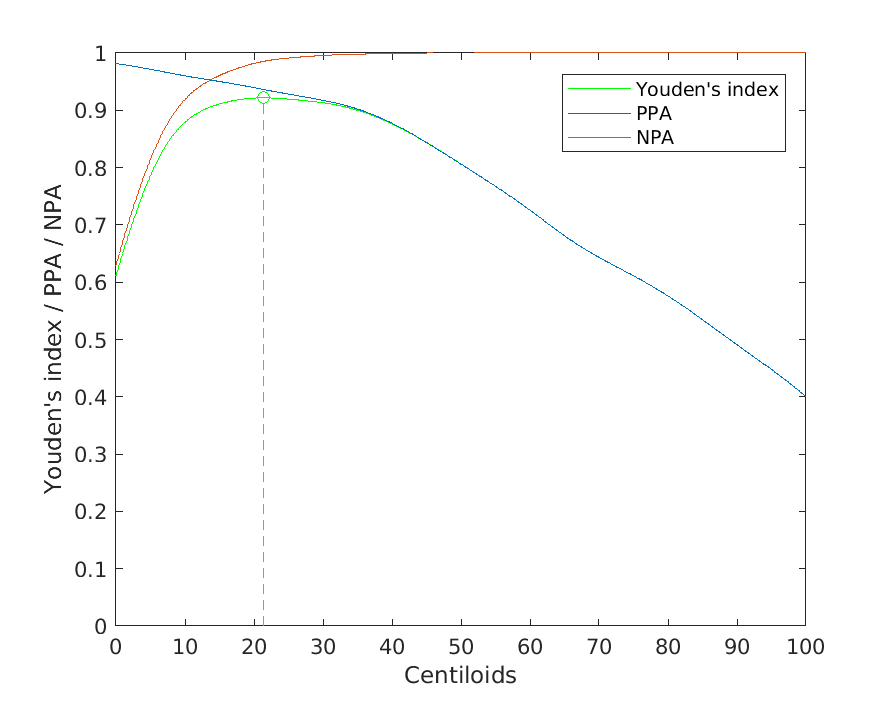 | 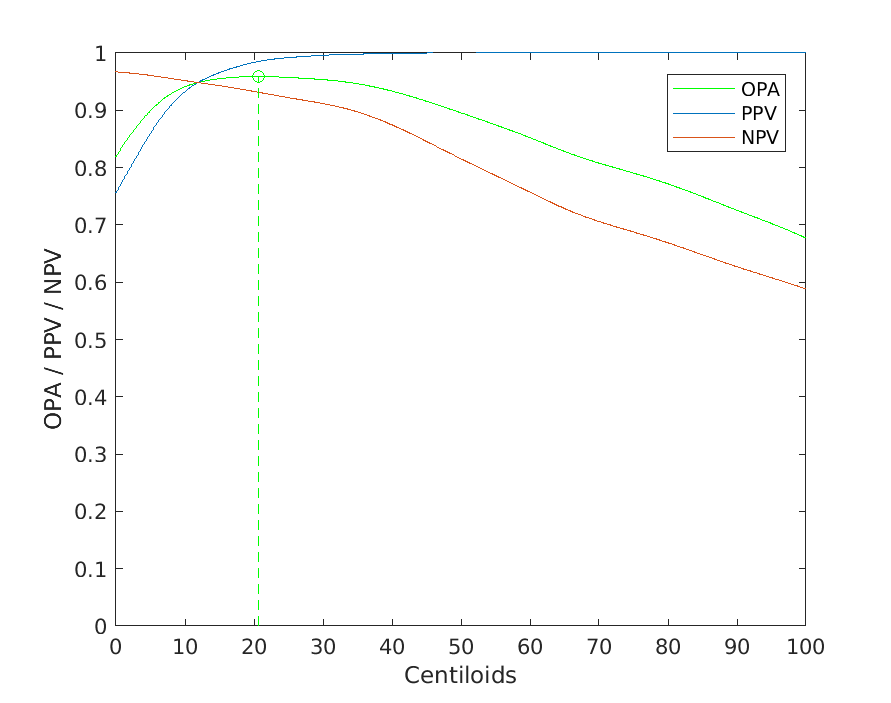 |
| **tTau/Aβ_42_** | 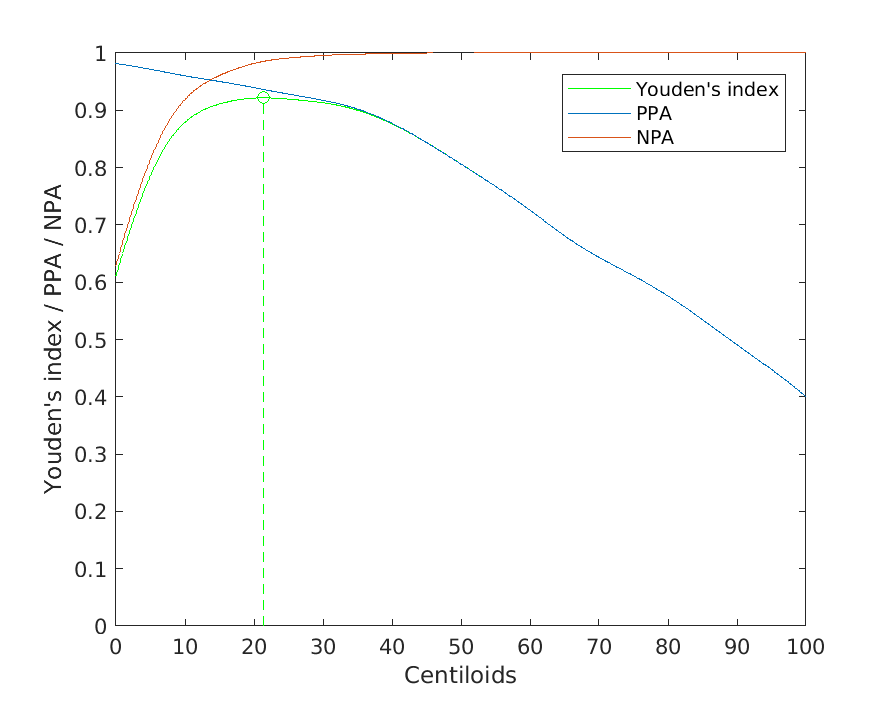 | 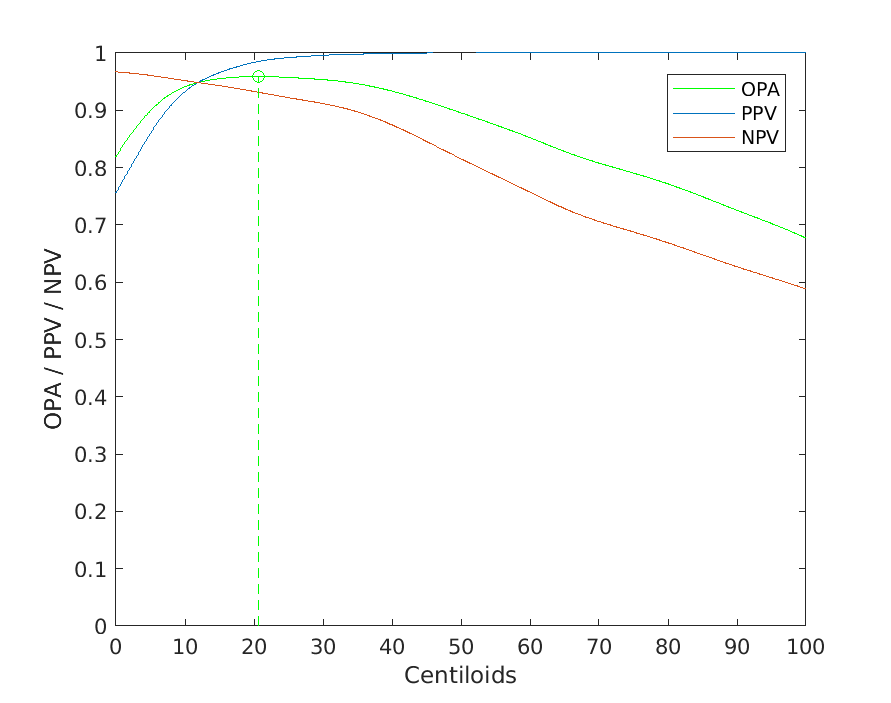 |
| **pTau** | 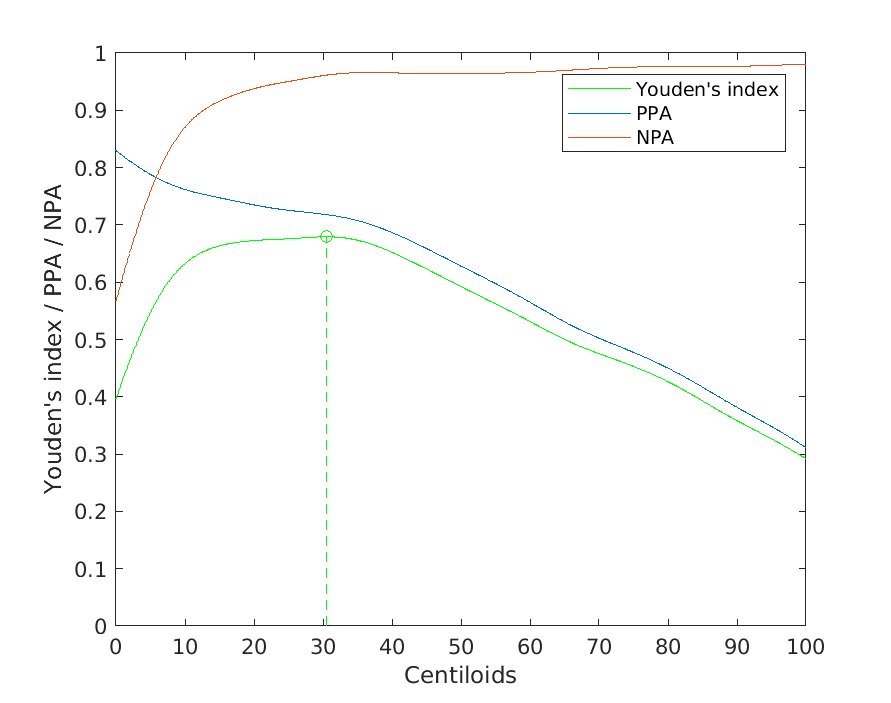 | 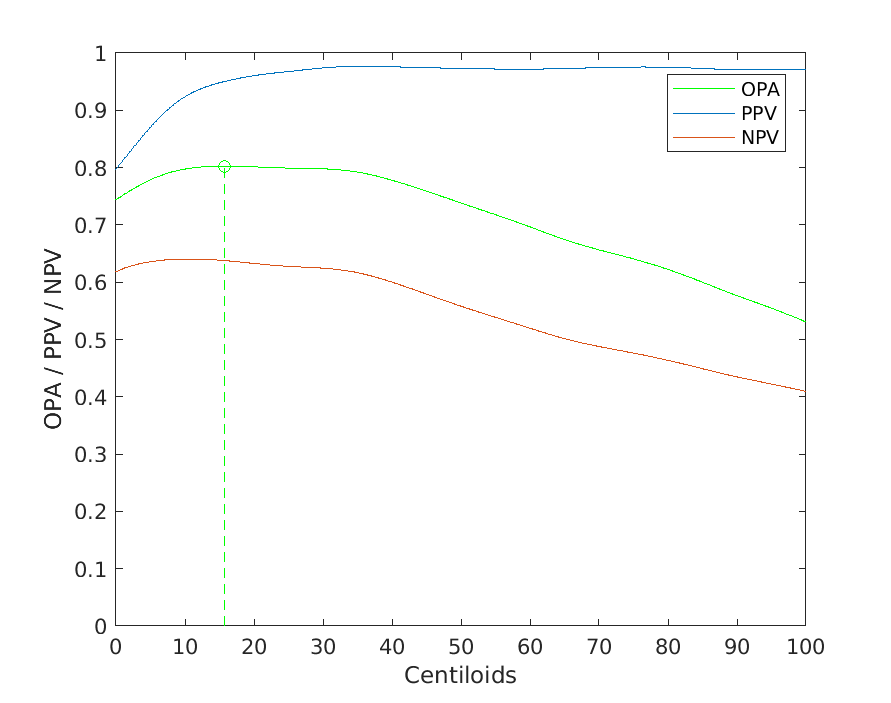 |
| **tTau** | 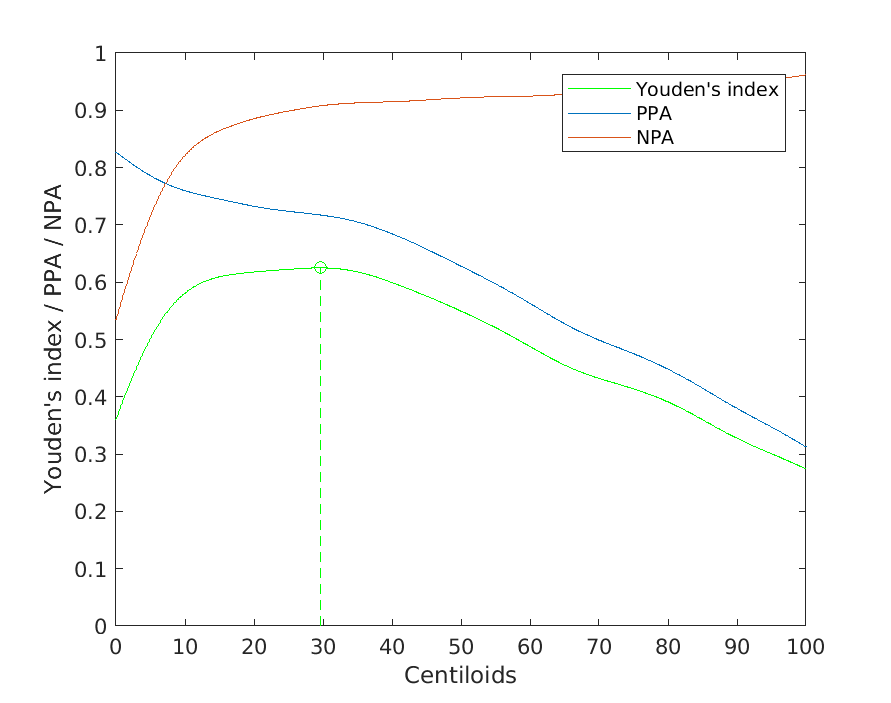 | 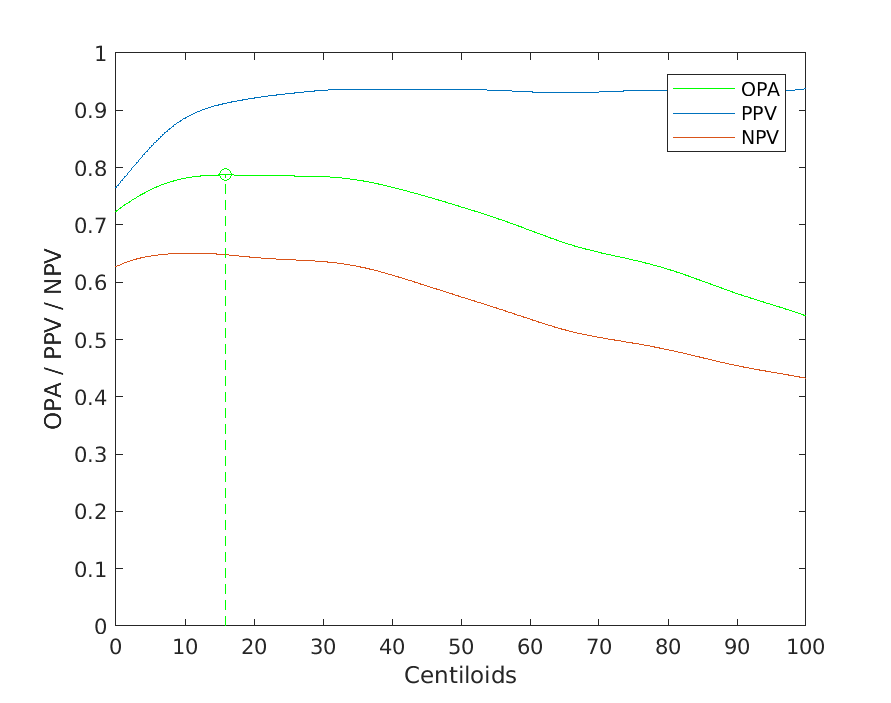 |

**Figure s2:** Derivation of amyloid PET Centiloid cut-offs against GZ threshold of Aβ_42_ (first row), pTau/Aβ_42_ (second row) and tTau/Aβ_42_ (third row), pTau (fourth row) and tTau (fifth row) CSF biomarkers. The Centiloid value that maximized YI (left column) or OPA (right column) was selected as the optimal cut-off. PPA and NPA are also shown (left column), and also PPV and NPV (right column). All participants' information was used to derive these cut-offs.

Abbreviations: GZ, grey zone; Aβ, amyloid; pTau, phosphorylated tau; tTau, total tau; CSF, cerebrospinal fluid; YI, Youden's Index; OPA, overall percent agreement ("accuracy); PPA, positive percentage agreement; NPA, negative percentage agreement, PPV, positive predictive value; NPV, negative predictive value.

|  | **Youden’s index** | **Overall percentage agreement** |
| --- | --- | --- |
| **Aβ_42_** | 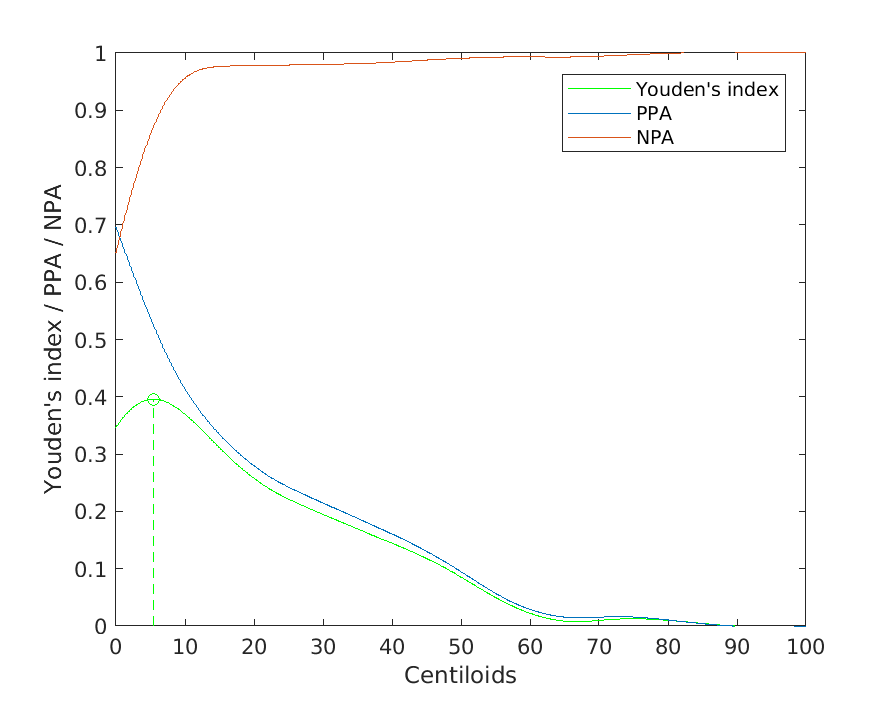 | 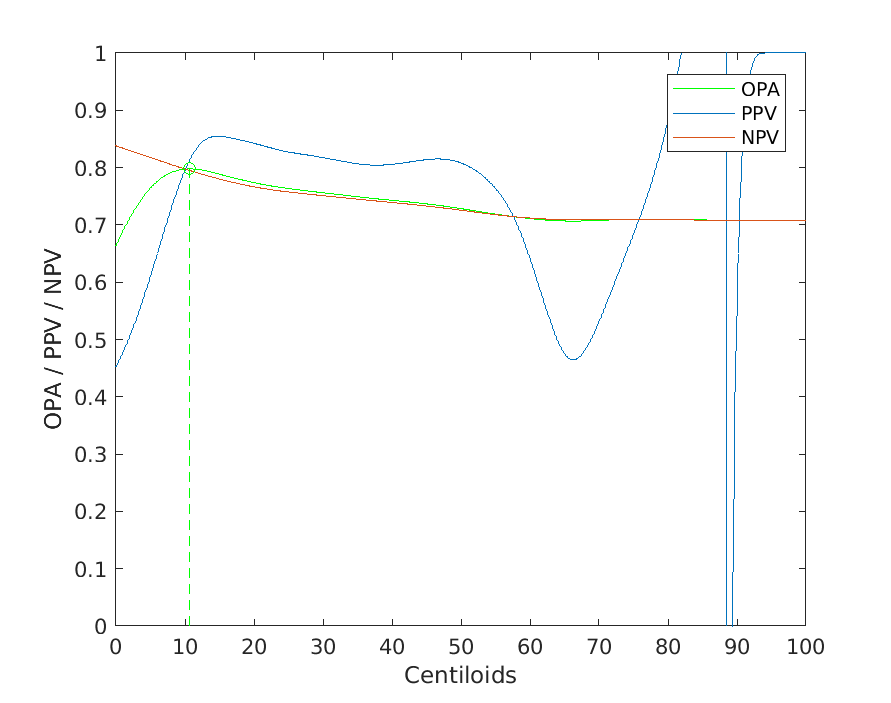 |
| **pTau/Aβ_42_** | 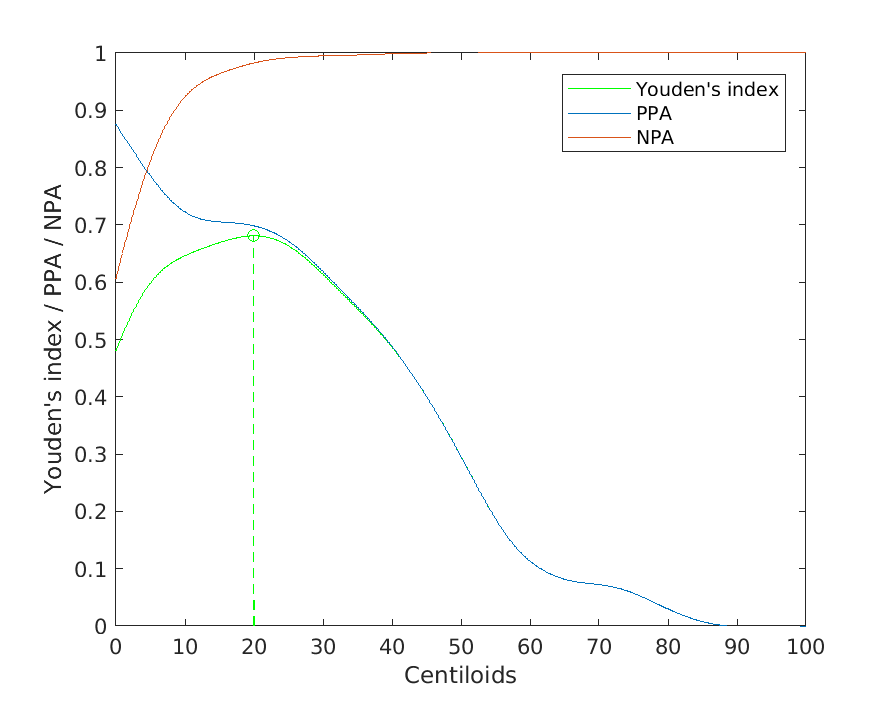 | 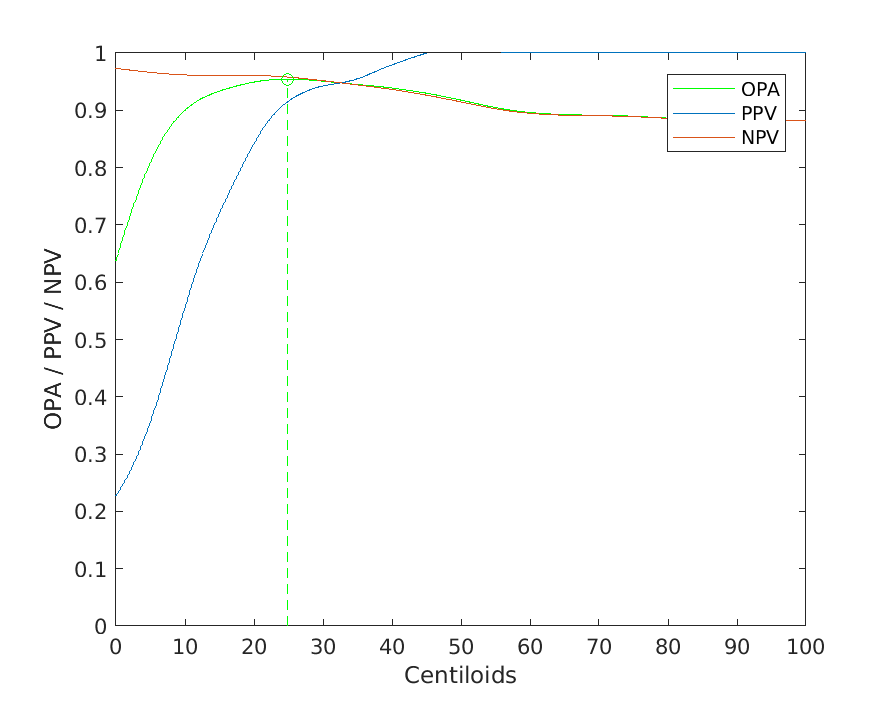 |
| **tTau/Aβ_42_** | 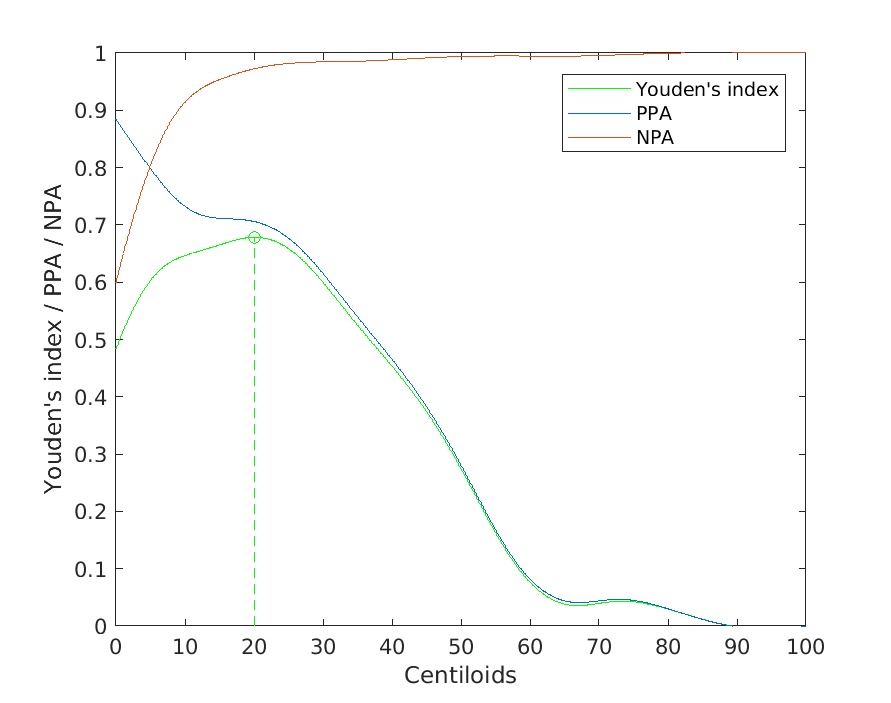 | 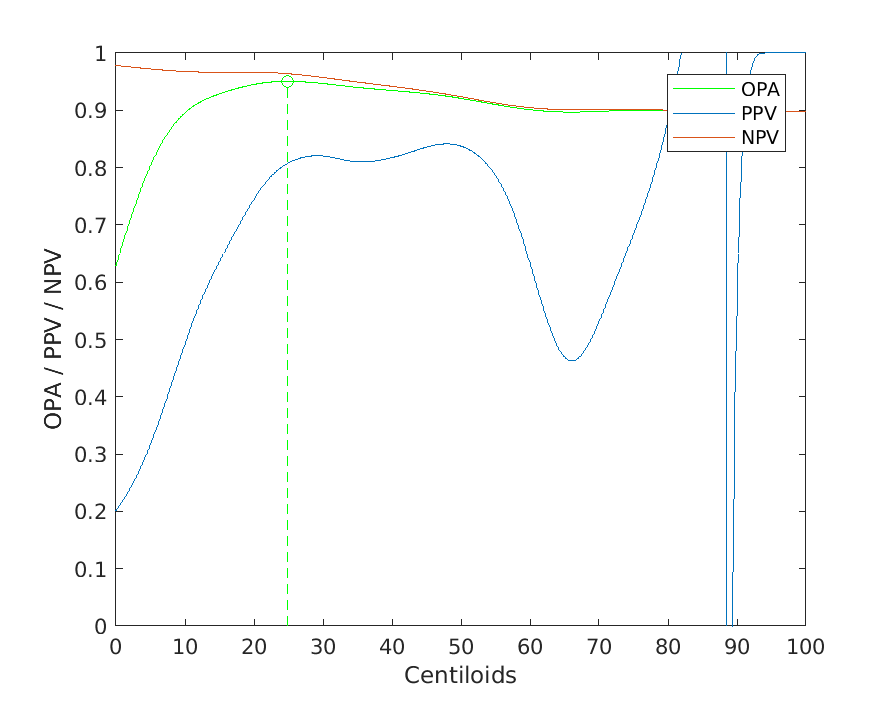 |
| **pTau** | 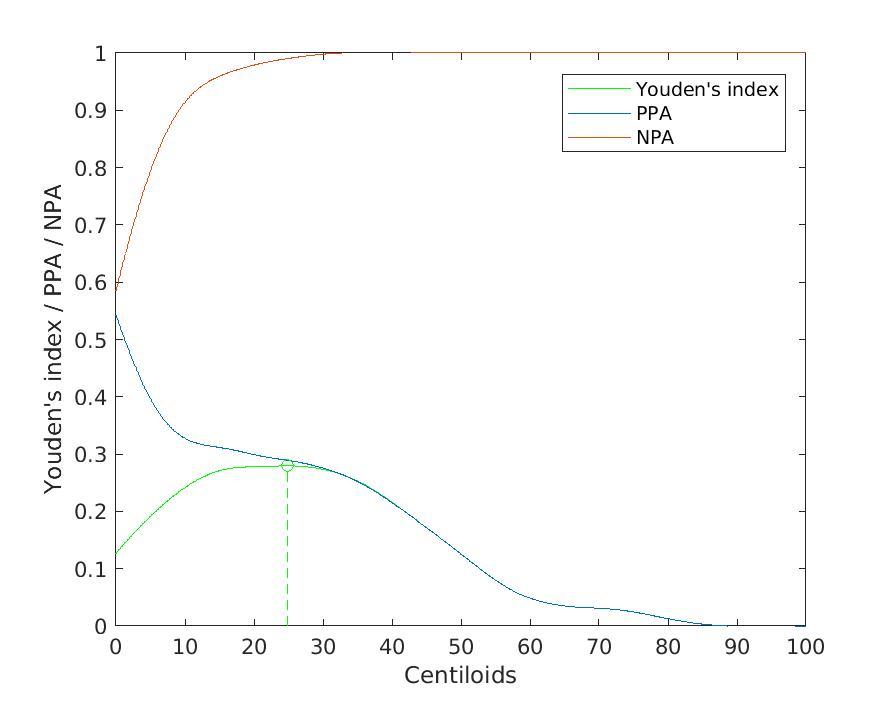 | 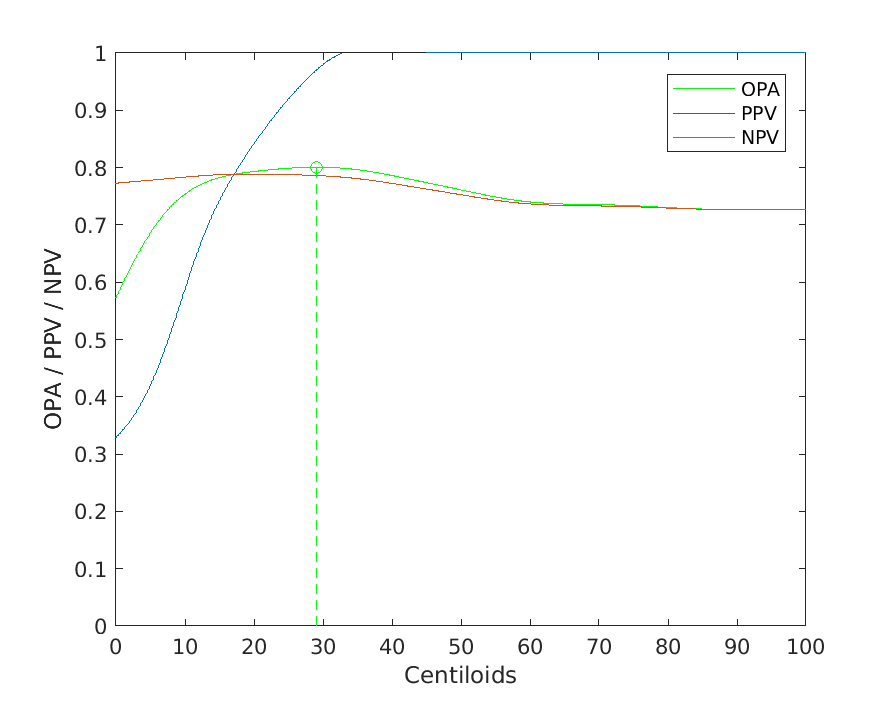 |
| **tTau** | 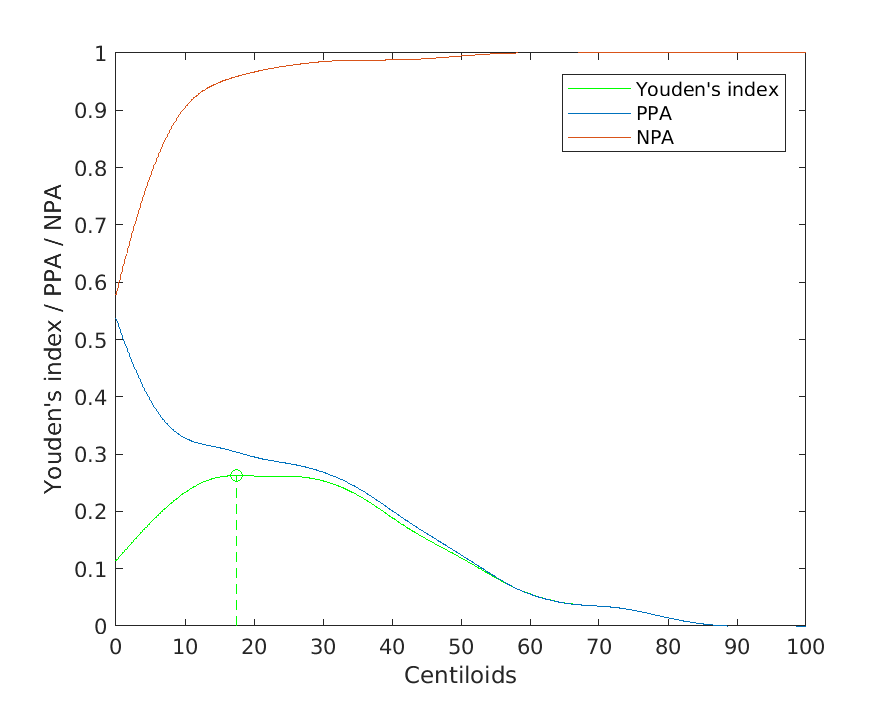 | 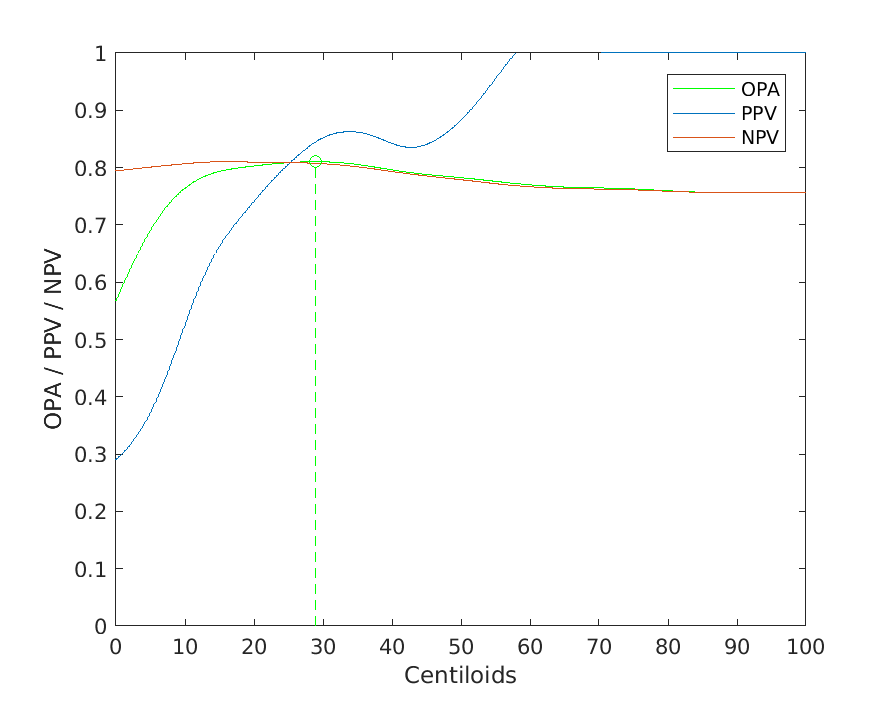 |

**Figure s3:** Derivation of amyloid PET Centiloid cut-offs on ALFA+ participants against Aβ_42_ (first row), pTau/Aβ_42_ (second row) and tTau/Aβ_42_ (third row), pTau (fourth row) and tTau (fifth row) CSF biomarkers. The Centiloid value that maximized YI (left column) or OPA (right column) was selected as the optimal cut-off. PPA and NPA are also shown (left column), and also PPV and NPV (right column). Only ALFA+ participants' information was used to derive these cut-offs.

Abbreviations: Aβ, amyloid; pTau, phosphorylated tau; tTau, total tau; CSF, cerebrospinal fluid; YI, Youden's Index; OPA, overall percent agreement ("accuracy); PPA, positive percentage agreement; NPA, negative percentage agreement; NPA, negative percentage agreement, PPV, positive predictive value; NPV, negative predictive value.

|  | **Youden’s index** | **Overall percentage agreement** |
| --- | --- | --- |
| **Aβ_42_** | 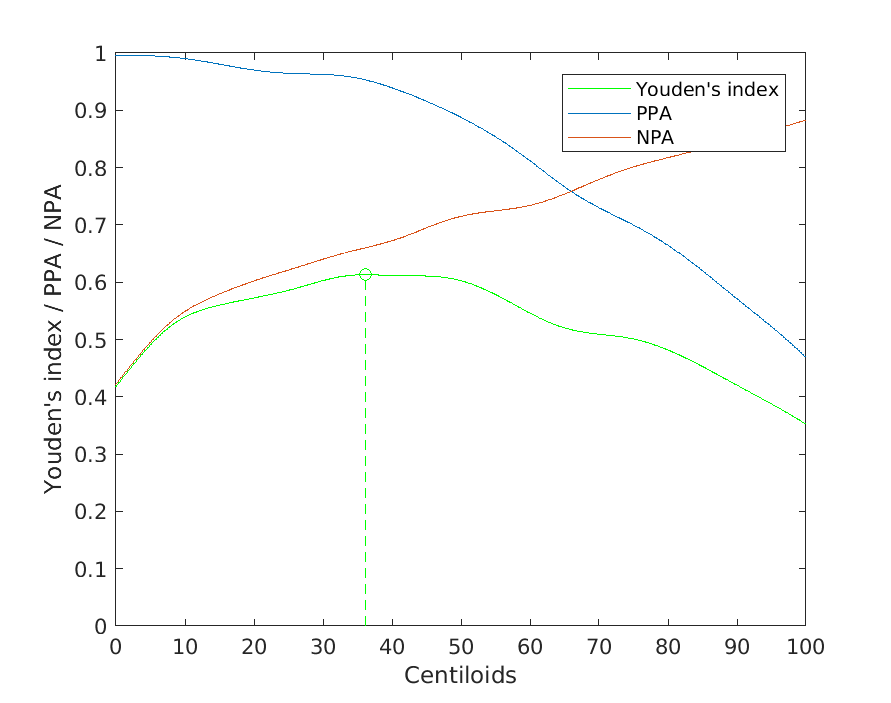 | 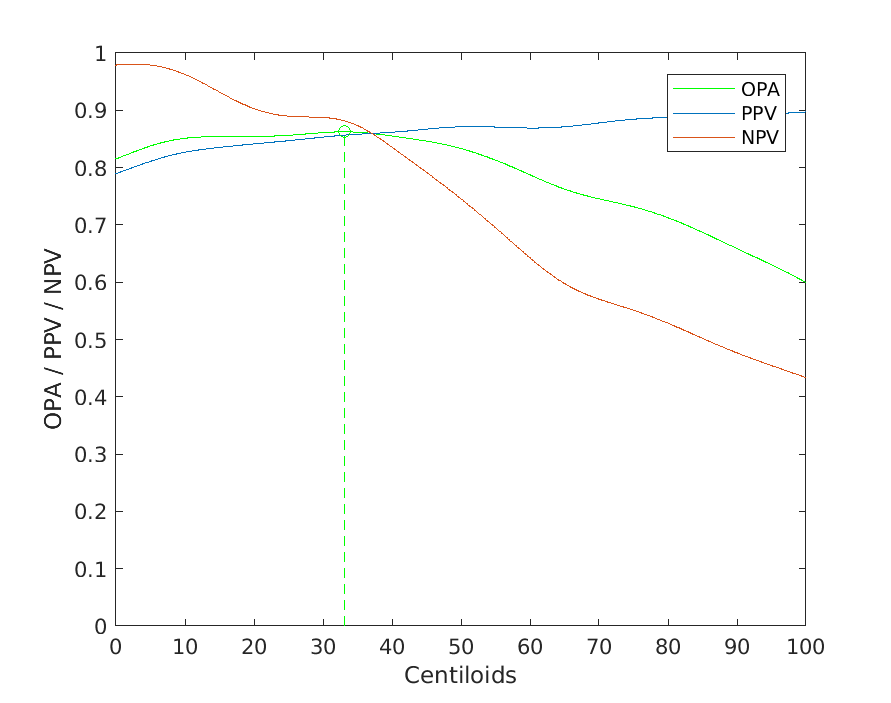 |
| **pTau/Aβ_42_** | 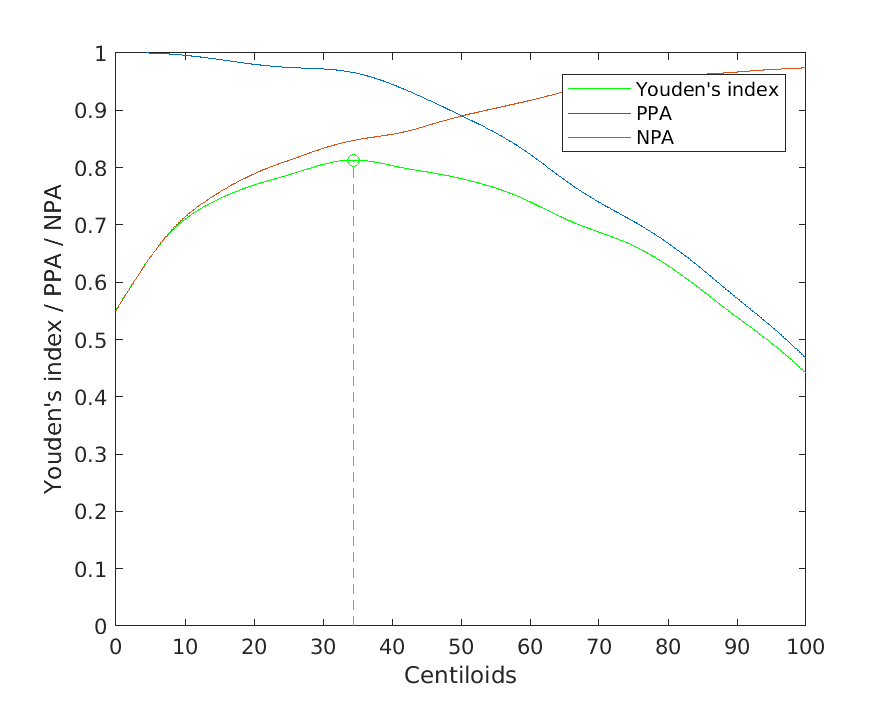 | 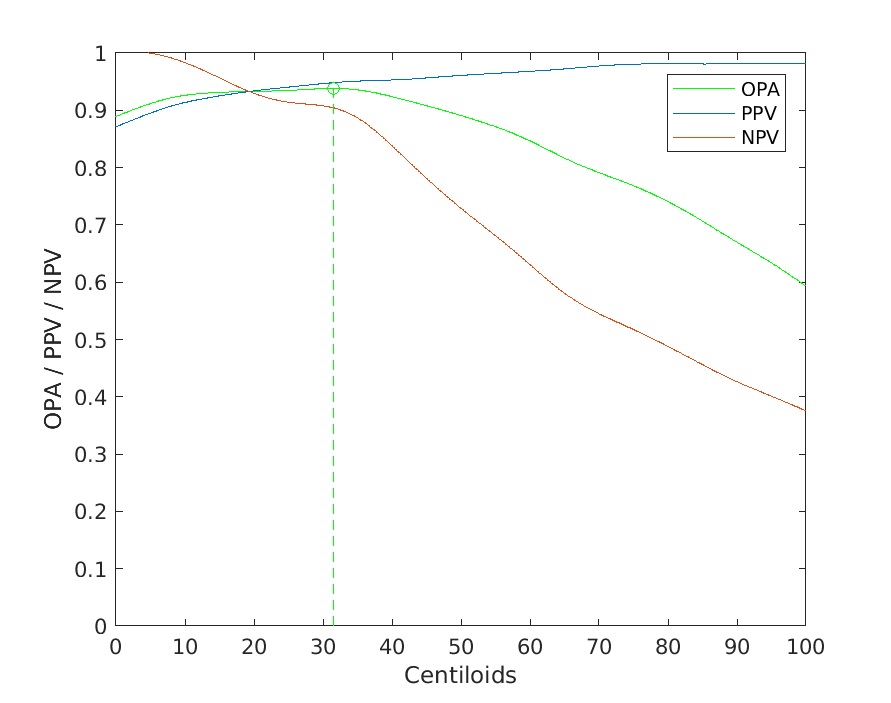 |
| **tTau/Aβ_42_** | 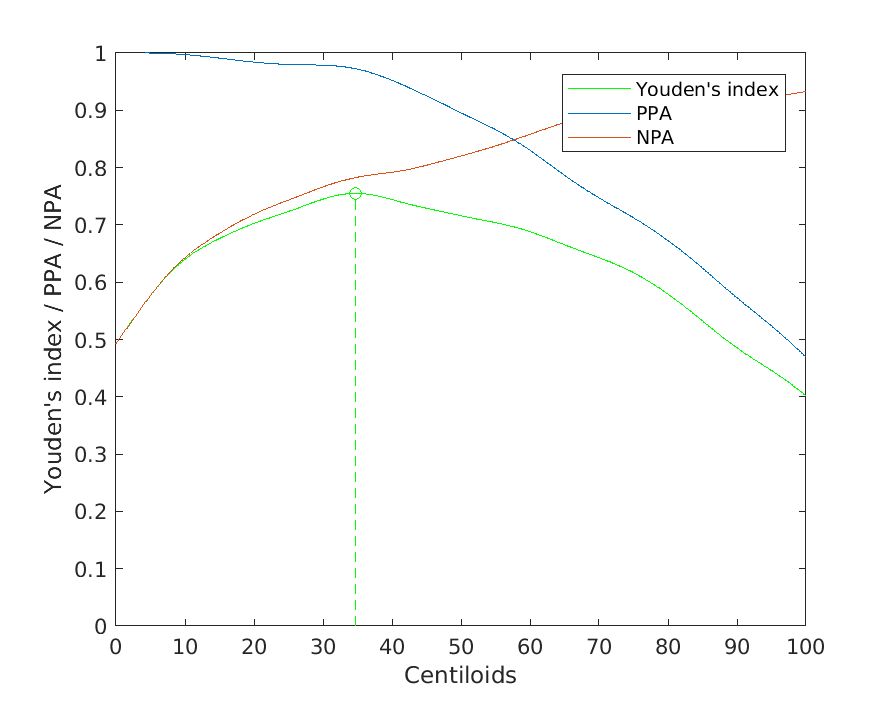 | 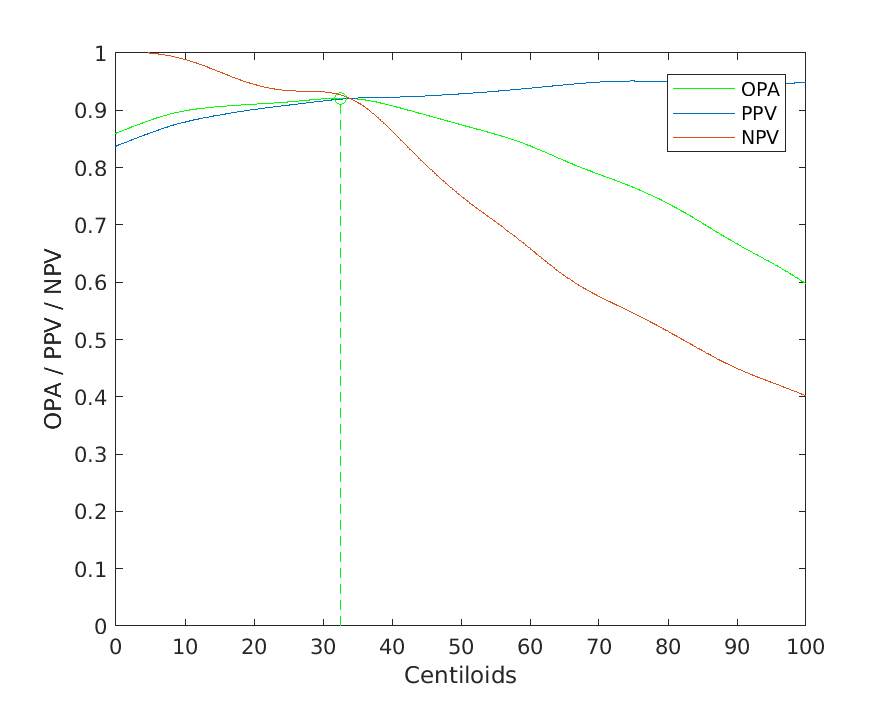 |
| **pTau** | 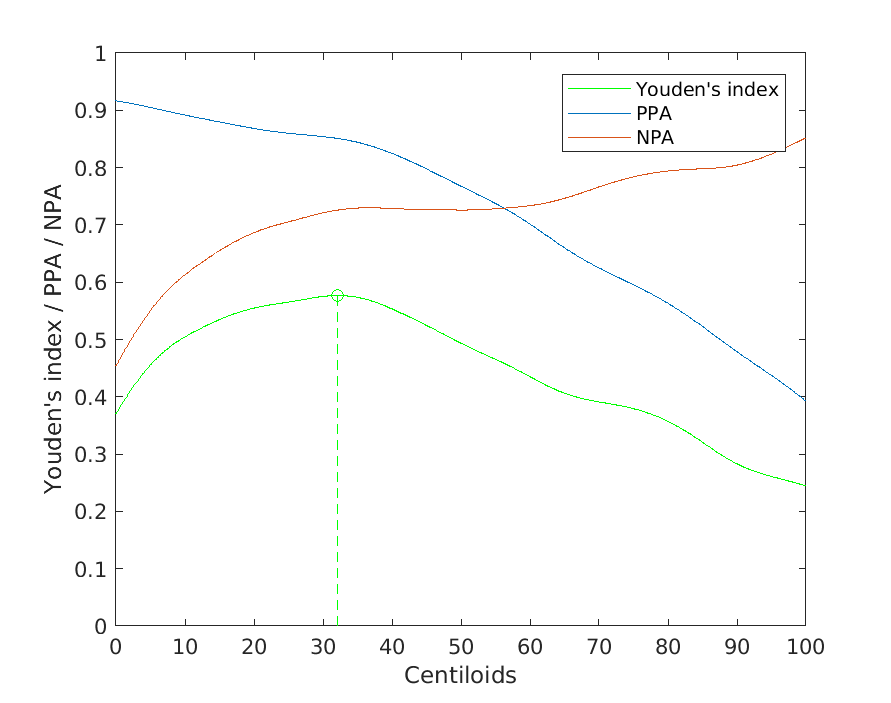 | 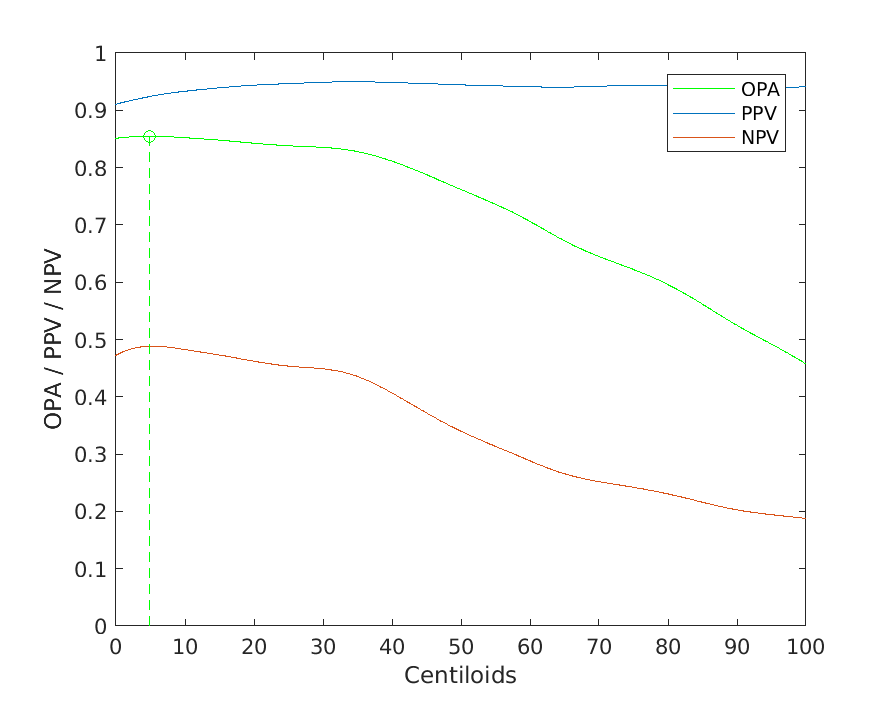 |
| **tTau** | 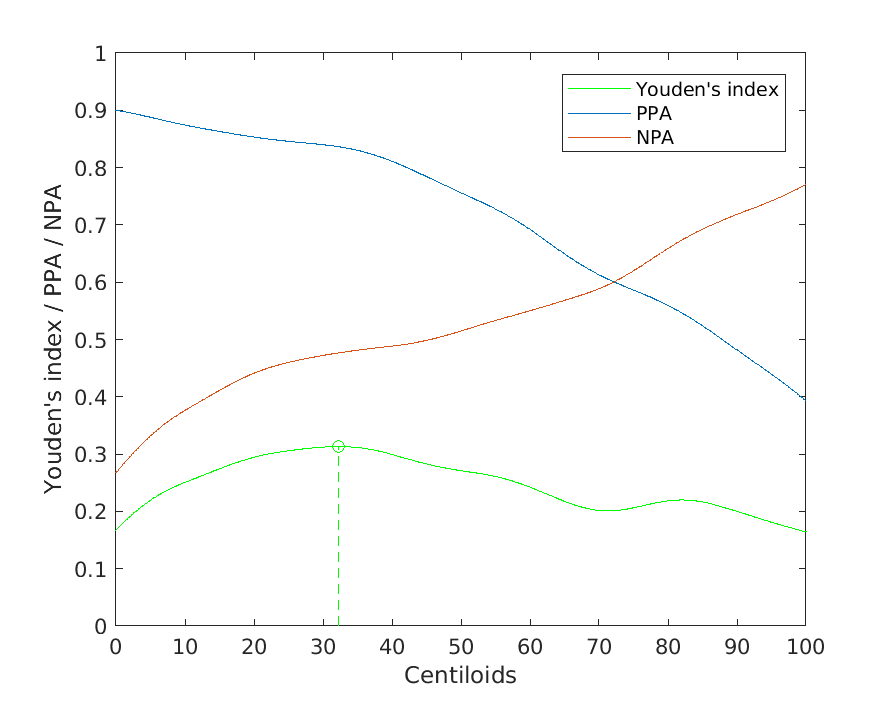 | 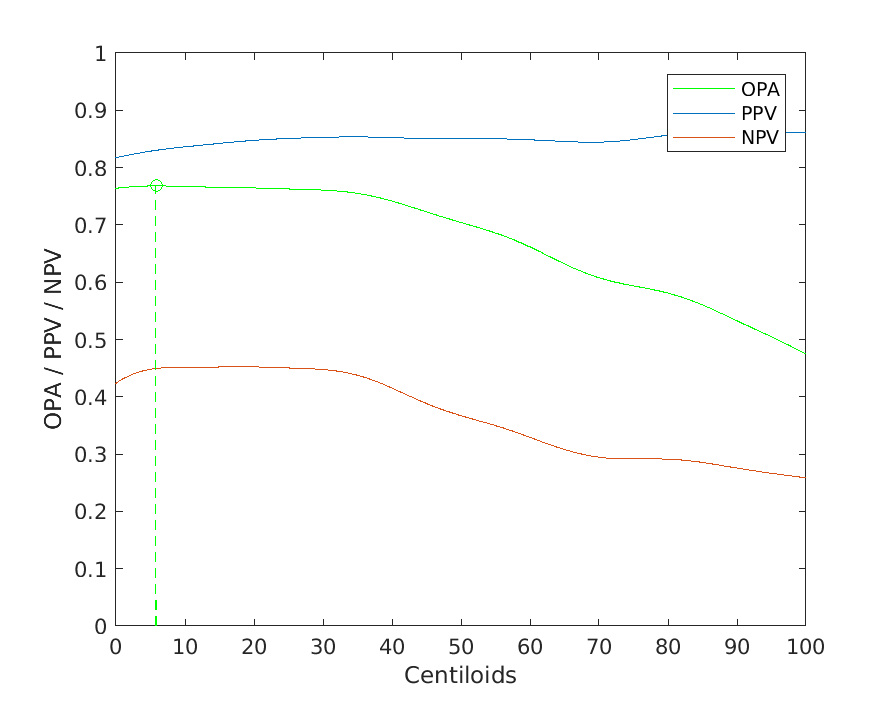 |

**Figure s4:** Derivation of amyloid PET Centiloid cut-offs on ADNI participants against Aβ_42_ (first row), pTau/Aβ_42_ (second row) and tTau/Aβ_42_ (third row), pTau (fourth row) and tTau (fifth row) CSF biomarkers. The Centiloid value that maximized YI and OPA was selected as the optimal cut-off (left column). PPA and NPA are also shown (right column). Only ADNI participants' information was used to derive these cut-offs.

Abbreviations: Aβ, amyloid; pTau, phosphorylated tau; tTau, total tau; CSF, cerebrospinal fluid; YI, Youden's Index; OPA, overall percent agreement ("accuracy); PPA, positive percentage agreement; NPA, negative percentage agreement; NPA, negative percentage agreement, PPV, positive predictive value; NPV, negative predictive value.

### References

1. Schindler SE, Gray JD, Gordon BA, Xiong C, Batrla-Utermann R, Quan M, et al. Cerebrospinal fluid biomarkers measured by Elecsys assays compared to amyloid imaging. Alzheimer’s Dement. Elsevier Inc.; 2018;1–10.

2. Hansson O, Seibyl J, Stomrud E, Zetterberg H, Trojanowski JQ, Bittner T, et al. CSF biomarkers of Alzheimer’s disease concord with amyloid-β PET and predict clinical progression: A study of fully automated immunoassays in BioFINDER and ADNI cohorts. Alzheimer’s Dement. 2018;1–12.

3. Molinuevo JL, Blennow K, Dubois B, Engelborghs S, Lewczuk P, Perret-Liaudet A, et al. The clinical use of cerebrospinal fluid biomarker testing for Alzheimer’s disease diagnosis: A consensus paper from the Alzheimer’s Biomarkers Standardization Initiative. Alzheimer’s Dement. 2014;10:808–17.

4. Klunk WE, Koeppe RA, Price JC, Benzinger TL, Devous MD, Jagust WJ, et al. The Centiloid project: Standardizing quantitative amyloid plaque estimation by PET. Alzheimer’s Dement. Elsevier Inc; 2015;11:1–15.e4.
